# The spatially resolved transcriptome signatures of glomeruli in chronic kidney disease

**DOI:** 10.1172/jci.insight.165515

**Published:** 2024-03-22

**Authors:** Geremy Clair, Hasmik Soloyan, Paolo Cravedi, Andrea Angeletti, Fadi Salem, Laith Al-Rabadi, Roger E. De Filippo, Stefano Da Sacco, Kevin V. Lemley, Sargis Sedrakyan, Laura Perin

**Affiliations:** 1 Biological Sciences Division, Pacific Northwest National Laboratory, Richland, Washington, USA.; 2 The GOFARR Laboratory, The Saban Research Institute, Division of Urology, Children’s Hospital Los Angeles, Los Angeles, California, USA.; 3 Department of Medicine, Translational Transplant Research Center, Icahn School of Medicine at Mount Sinai, New York, New York, USA.; 4 Nephrology Dialysis and Renal Transplantation, IRCCS Istituto Giannina Gaslini, Genova, Italy.; 5 Department of Laboratory Medicine and Pathology, Mayo Clinic, Jacksonville, Florida, USA.; 6 Division of Nephrology and Hypertension, Department of Internal Medicine, University of Utah Health, Salt Lake City, Utah, USA.; 7 Department of Urology, Keck School of Medicine, and; 8 Division of Nephrology, Department of Pediatrics, University of Southern California, Los Angeles, California, USA.

**Keywords:** Nephrology, Chronic kidney disease

## Abstract

Here, we used digital spatial profiling (DSP) to describe the glomerular transcriptomic signatures that may characterize the complex molecular mechanisms underlying progressive kidney disease in Alport syndrome, focal segmental glomerulosclerosis, and membranous nephropathy. Our results revealed significant transcriptional heterogeneity among diseased glomeruli, and this analysis showed that histologically similar glomeruli manifested different transcriptional profiles. Using glomerular pathology scores to establish an axis of progression, we identified molecular pathways with progressively decreased expression in response to increasing pathology scores, including signal recognition particle–dependent cotranslational protein targeting to membrane and selenocysteine synthesis pathways. We also identified a distinct signature of upregulated and downregulated genes common to all the diseases investigated when compared with nondiseased tissue from nephrectomies. These analyses using DSP at the single-glomerulus level could help to increase insight into the pathophysiology of kidney disease and possibly the identification of biomarkers of disease progression in glomerulopathies.

## Abstract 2

Digital spatial profiling reveals distinct glomerular transcriptional signatures of Alport syndrome, focal segmental glomerulosclerosis, and membranous nephropathy.

## Graphical abstract

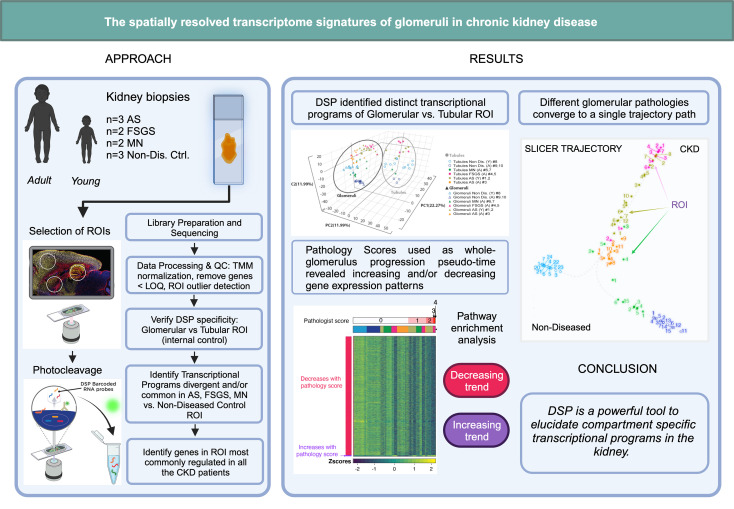

## Introduction

The growing prevalence of chronic kidney disease (CKD) and its economic burden is a global health problem. There is an unmet need for better understanding of the pathophysiology of glomerular diseases, which represent a significant cause of CKD, and the underlying mechanisms of disease progression (1, 2). The glomerulus, the filtration unit of the kidney, comprises 3 specialized cell types; the podocyte and the glomerular endothelial cell (GEC), which are separated by a glomerular basement membrane (GBM), along with the mesangial cell, all of which contribute to glomerular function (3, 4). Intercellular communication and proper interaction with the GBM are key to maintaining the integrity of the filtration apparatus. Even small disturbances in gene expression, like, for example, changes in VEGF or hypoxia-inducible factor-1 expression, can lead to the development of various glomerulopathies (4, 5).

Different transcriptomic platforms, including bulk RNA-Seq (6, 7), single-cell RNA-Seq (scRNA-Seq) (8), and single-nucleus RNA-Seq (snRNA-Seq) (9), have allowed characterization of kidney cell subtypes and helped uncover new molecular pathways responsible for disease progression, but the relation between these transcriptional data and their spatial localization within the kidney is still unclear. Without this context, interactions between cells and their localization in different tissue compartments can only be inferred or validated with low-throughput imaging assays. Spatially resolved whole-genome analysis, which encompasses the combination of traditional high-throughput quantitative transcriptomics and high-resolution tissue imaging, by contrast, can be used to correlate specific tissue structures with changes in gene expression, providing spatial transcriptomic maps, leading to an unbiased characterization of tissue niches and cellular compartments (10).

In this study, using the whole-transcriptome GeoMx digital spatial profiling (DSP) platform (11), we assessed the spatially resolved transcriptome changes in human kidney glomeruli derived from biopsies from patients affected by 1 of 3 glomerular diseases: Alport syndrome (AS), focal segmental glomerulosclerosis (FSGS), and membranous nephropathy (MN); we used glomeruli from specimens of nondiseased kidneys for comparison. AS is a monogenic kidney disease caused by mutations in *COL4A3*, *COL4A4*, or *COL4A5* genes, resulting in impairment of the GBM due to the failure to assemble normal ColIVα3α4α5 trimers (12–14). FSGS describes a pathological condition found in various kidney diseases, marked by initial segmental scarring of the glomerular tuft (15–17). MN is an immunologically mediated disease caused by the deposition of immune complexes within the filtration barrier, leading to inflammation and podocyte damage (18, 19). Despite these quite different etiologies, these kidney diseases all cause glomerular scarring and loss of podocytes and, in many cases, progress to kidney failure.

In this work, to reveal common pathways that drive pathological processes common to all 3 diseases, we compared the sequencing data for each glomerulus to its corresponding pathology score by linear regression analysis (20) and correlated increasing pathology scores to a specific set of genes with increasing or decreasing expression. Next, to determine the transcriptional distances between glomeruli and characterize disease progression, we used SLICER trajectory analysis (21), without relying on the pathology scores as an unbiased approach, followed by a postanalysis comparison of trajectories with pathology scores. We also performed a correlation analysis using established glomerular cell–specific markers to identify a panel of genes that positively correlated with one or more of the podocyte genes (*NPHS1*, *NPHS2*, *WT1*) or with GEC genes (*EHD3*, *CDH5*, *TEK*) or with mesangial cell genes *(PDGFRB*, *GATA3*, *CD44*). Our analysis also identified a transcriptional signature comprising genes involved in pathways, such as signal recognition particle–dependent (SRP-dependent) cotranslational protein targeting to membrane and selenocysteine synthesis, as well as genes such as *ADAMTS13*, *GJA5*, and *CCDN1* present in all diseased glomeruli, regardless of disease type or the extent of pathology.

Defining transcriptional programs at the single-glomerulus level is a powerful way of gaining insight into the pathophysiology of kidney disease, with the potential for clarifying biological processes key to understanding mechanisms of progression that have been largely unexplored due to the limitations of other gene profiling techniques, in addition to allowing the identification of genes and pathways that could represent promising targets for new CKD therapeutic strategies.

## Results

### Tissue histopathology and quality control assessment of DSP RNA detection.

**Figure 1:**
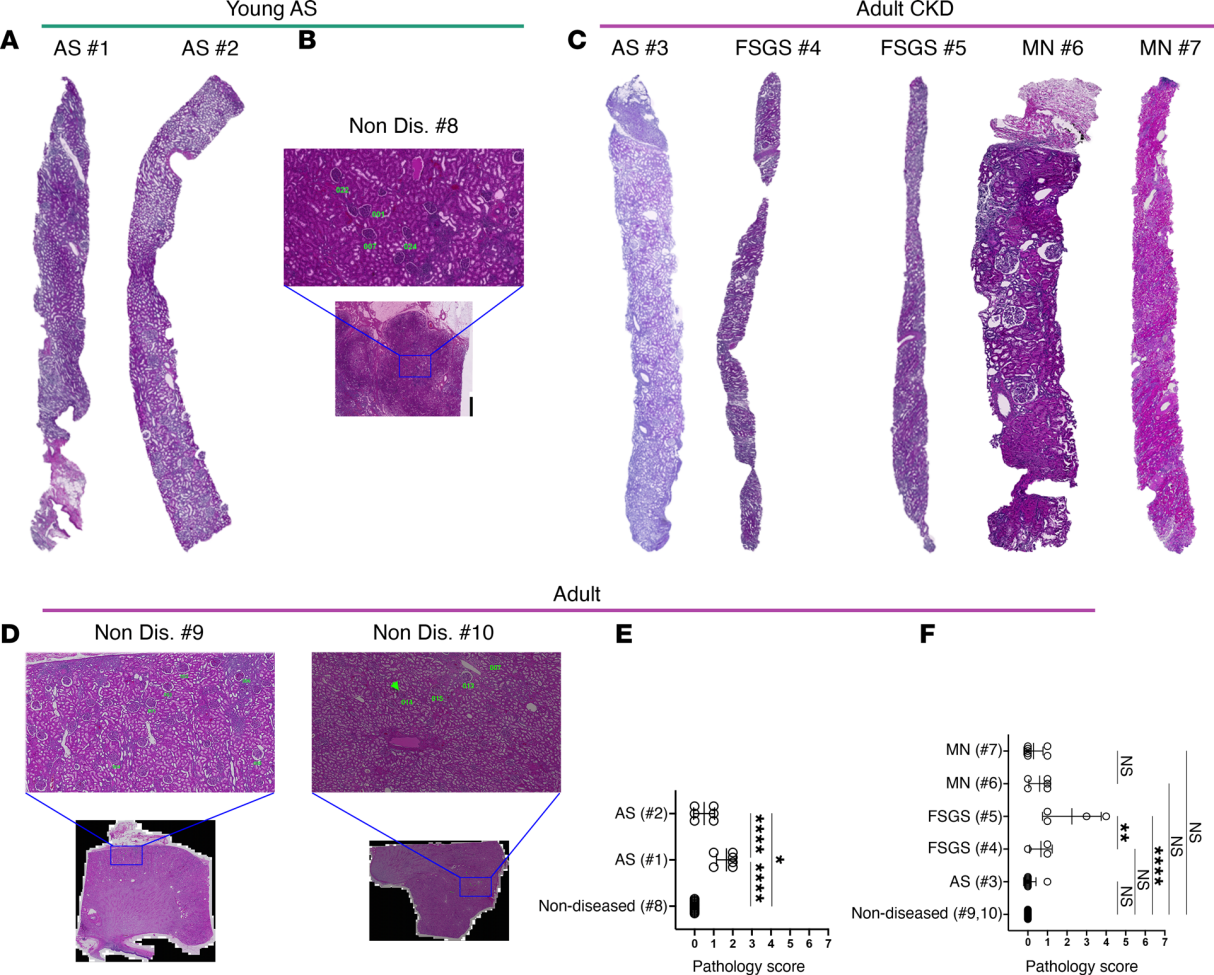
Clinical features and histopathology of glomeruli from patients with AS, FSGS, and MN as well as individuals with nondiseased glomeruli. (**A**–**D**) Representative histology micrographs from serially sectioned kidney biopsies from a 9-year-old patient with AS (no. 1 and 2; **A**); a 6-year-old patient without disease (no. 8; **B**); a 25-year-old patient with AS (no. 3), 21- and 25-year-old patients with FSGS (no. 4 and 5), 21- and 62-year-old patients with MN (no. 6 and 7) (**C**); and 56-year-old and approximately 40-year-old patients without disease (non-diseased; no. 9 and 10) (original magnification, ×20). Select glomeruli (the same glomeruli also analyzed by DSP) were assessed histopathologically. Blown up projections in **B** and **D** show higher-magnification (original magnification, ×20) images of the tissue sections from where the glomerular ROIs were selected for DSP. (**E** and **F**) H&E-stained histology slides of biopsy samples from patients with AS (no. 1–3), FSGS (no. 4 and 5), and MN (no. 6 and 7) and samples without disease (no. 8, 9, and 10) were scored by a kidney pathologist in a blinded manner. Dot plots depicting the glomerular pathology scores in kidney biopsies from AS (no. 1 and 2) and nondiseased control glomeruli (no. 8) (**E**), and patients with AS (no. 3), FSGS (no. 4 and 5), and MN (no. 7, 6) and nondiseased glomeruli (**F**). *P < 0.05; **P < 0.01; ****P < 0.0001. Data are shown as the mean ± SD.

**Figure 2:**
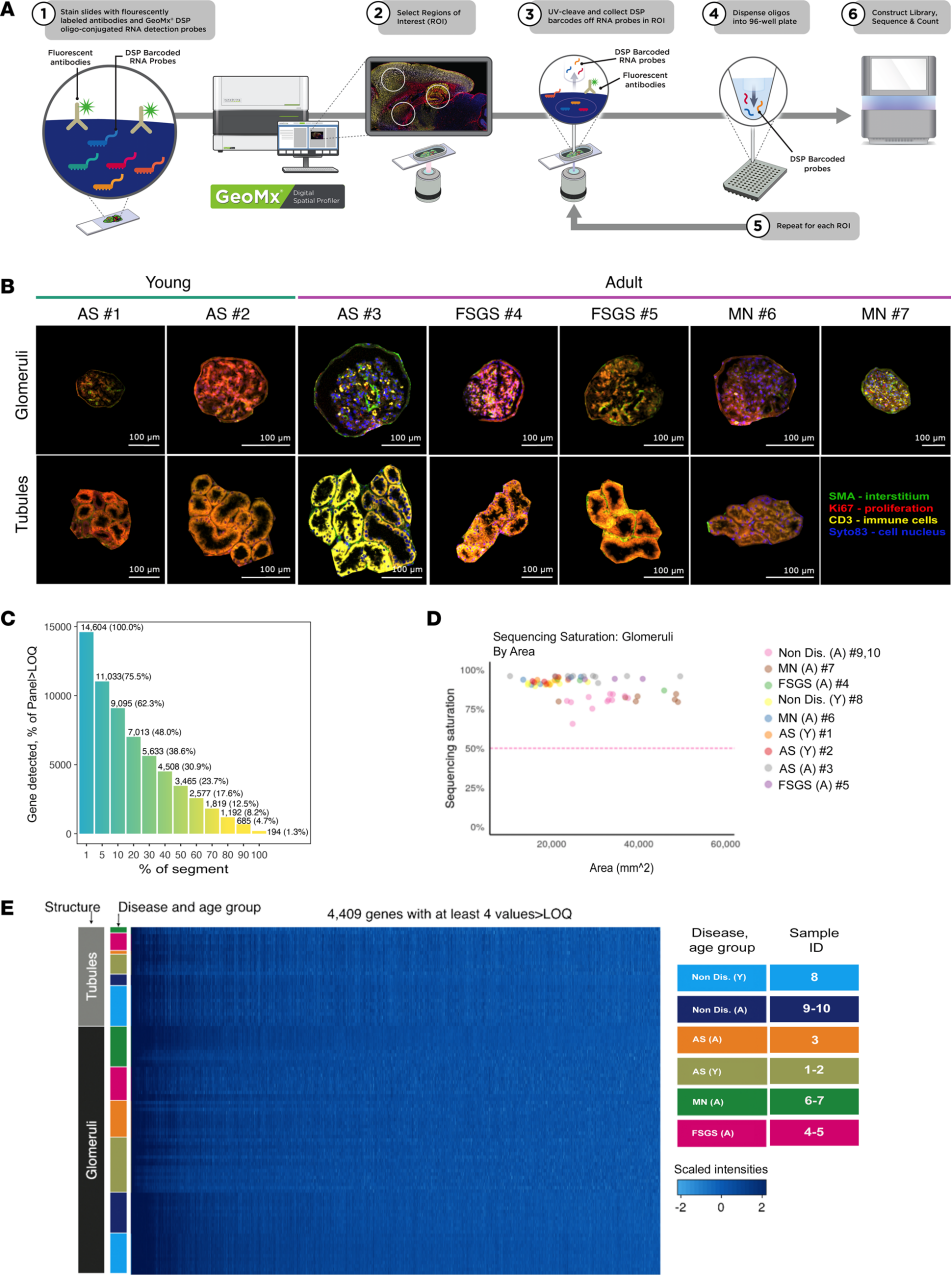
GeoMx digital spatial profiling platform workflow, selection of glomerular and tubular ROI, and quality control assessment. (**A**) Schematic illustration of the Nanostring GeoMx Digital Spatial Profiler workflow for interrogating multiple RNA analytes from a single paraffin-embedded tissue section. Analytes in the tissue section are conjugated with oligo tags via photocleavable linker, and glomerular ROIs are defined with the aid of morphology markers. Spatially mapped UV illumination allows oligo tags to be released from the analyte into a 96-well plate. The collected oligos are then subject to sequencing to obtain digital counts per ROI. (**B**) Select scans of glomerular and tubular ROI representative of AS (no. 1–3), FSGS (no. 4 and 5), and MN (no. 6 and 7) were immunostained for smooth muscle actin (SMA, green), Syto83 (nucleic acid stain, blue), CD3 (T cell marker, yellow), and Ki67 (marker of cellular proliferation, red) to guide the selection of ROIs. Individual glomeruli and tubules in the DSP were manually defined as individual geometric segments, with sizes ranging from 9,700 to 168,000 mm^2^. Scale bar: 100 μm. (**C**) Histogram showing the percentage of genes detected above LOQ relative to the percentage of glomerular segments analyzed. The number of genes detected per percentage of segments is depicted on top of each bar. (**D**) Dot plot depicting sequencing saturation (ranging between 75% and 95%) calculated over the areas of the glomerular ROIs. A, adult; Y, young. (**E**) Heatmap of TMM-normalized counts of transcripts, depicting the dynamic range of gene expression between glomerular and tubular ROI across all biopsies.

Glomeruli in all the biopsies (Table 1) were blindly scored by a kidney pathologist and compared with glomeruli (*n* = 24) from age-matched nondiseased tissue sections derived from partial nephrectomy specimens. Histopathology revealed variability in the diseased glomeruli, with some defined as normal and others showing various degrees of abnormality (Figure 1). To perform the GeoMx DSP Whole Transcriptome Assay (WTA), we followed the workflow established by Nanostring (Figure 2A), and we manually selected the regions of interest (ROIs) and the glomeruli, guided by immunofluorescence markers CD3, SMA, Ki67, and Syto83; these markers visibly allowed the identification of the different renal cells/structures (Figure 2B) by the NanoString GeoMx Digital Spatial Profiler. We also selected ROIs that identified tubules that were used as a quality control in our analysis to establish transcriptomic signatures of glomeruli compared with tubules, thus confirming validity of our selection. Following selection of 75 glomerular and 31 tubular ROIs, we performed RNA-Seq to explore compartment-specific transcriptional programs. Of note, we analyzed by DSP all the glomeruli in the diseased biopsies except those that did not contain enough cells for sequencing and, therefore, could not pass the quality control. We also analyzed histologically normal glomeruli selected arbitrarily in specimens from the nondiseased tissue (see Methods for selection criteria).

Consistent expression of genes above the limit of quantification (LOQ) was observed in many glomerular ROIs. For 50% of segments, 23.7% of the panel or 3,465 genes were detected above LOQ (Figure 2C). The sequencing saturation ranged between 75% and 95%, ensuring adequate assay sensitivity (Figure 2D), and the signal dynamic range was satisfactory (Figure 2E). Based on robust Mahalanobis distance (rMd) test, 2 glomerular and 2 tubular ROIs were eliminated from downstream analysis, as described in Methods (Supplemental Figure 1, A–C; supplemental material available online with this article; https://doi.org/10.1172/jci.insight.165515DS1); all the glomerular ROI analyzed by DSP are represented in Supplemental Figure 2.

**Figure 3:**
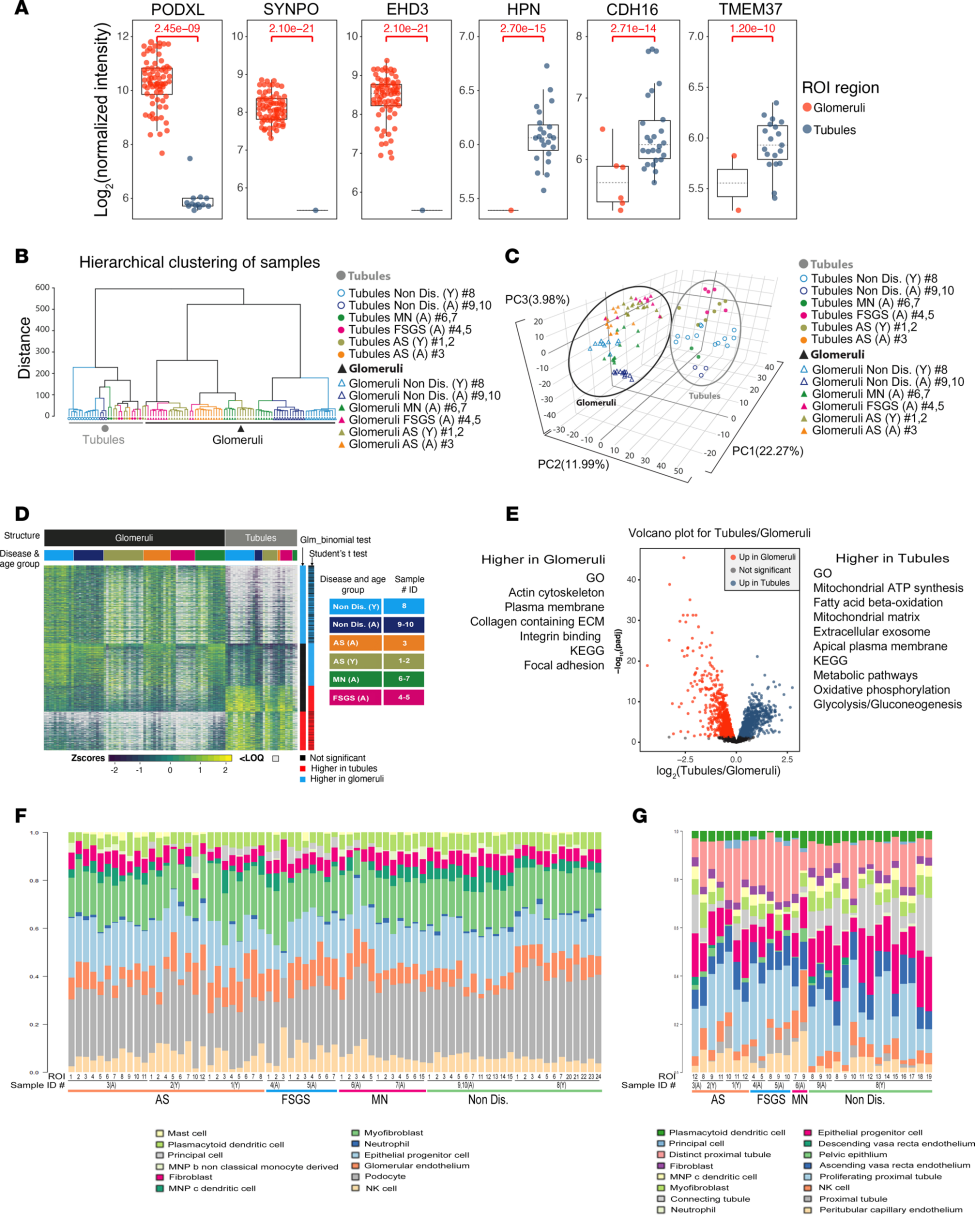
Gene expression and cell deconvolution analysis of glomerular and tubular ROIs. (**A**) Box plots of normalized intensities for *PODXL*, *SYNPO*, and *EDH3* (glomeruli-specific genes) as well as *HPN*, *CDH16*, and *TMEM37* (tubule-specific genes). Each dot represents either a glomerular (orange) or tubular (blue) ROI. Binomial GLM adjusted *P* values are indicated in red. (**B**) Dendrogram showing hierarchical clustering and transcriptional link between glomeruli (all samples) and tubules (all samples). A, adult; Y, young. (**C**). Unsupervised principal component analysis based on label-free quantification of the transcripts expressed in glomerular versus tubular ROIs in all kidney tissue segments analyzed based on principal components (PC1, PC2, PC3) constructed to capture the most variation in the samples. Percentage of total variance is indicated after each principal component. (**D**) Heatmap depicting the transcripts significantly modulated between glomerular and tubular ROIs in all kidney tissue segments analyzed (Student’s *t* test; binomial GLM test, Benjamini-Hochberg-adjusted [BH-adjusted] *P* < 0.05). Transcripts less than LOQ in value are shown in white. (**E**) Volcano plot representing the results of the Student *t* test (BH-adjusted *P* < 0.05) comparison of differential gene expression between glomerular and tubular ROIs in all the biopsies. A select list of GO terms and KEGG pathways significantly enriched (EASE-modified Fisher’s exact, *P* < 0.05) for genes upregulated in both glomeruli and tubules (Student’s *t* test and binomial test) is depicted on each side of the volcano plot. (**F** and **G**) Cell deconvolution analysis showing the abundance of cell types in glomerular ROIs (*n* = 73, **F**) and tubular ROIs (*n* = 29, **G**) from AS (no. 1–3), FSGS (no. 4 and 5), and MN (no. 7, 6) biopsies and their corresponding nondiseased controls, based on publicly available single-cell RNA-Seq experiments compiled into profile matrices by aggregating gene-wise counts of all annotated cell types (61).

To confirm specificity of our ROI selection method, we compared the transcriptional program between the glomerular and tubular ROIs across all samples. Glomerulus-specific genes, including *PODXL*, *SYNPO*, and *EHD3*, were expressed in higher abundance in glomerular ROIs; conversely, genes typical of the tubule segments of the nephron, including *HPN*, *CDH16*, and *TMEM37*, were highly enriched in tubular ROIs (Figure 3A). The transcriptional programs of glomeruli and tubules showed distinct clustering by hierarchical clustering analysis (Figure 3B) and separated clearly in principle component analysis (PCA; Figure 3C). Many genes highly expressed in glomeruli were mostly not detected in the tubules and vice versa (Figure 3D). We identified 2,664 genes that were differentially expressed (DE) between glomerular and tubule ROIs. Genes upregulated in the glomeruli were highly enriched in Gene Ontology (GO) terms and Kyoto Encyclopedia of Genes and Genomes (KEGG) pathways associated with cytoskeleton, cellular adhesion, and extracellular matrix (ECM); in contrast genes upregulated in tubules were most highly enriched in metabolic processes, including glycolysis and mitochondrial β-oxidation (Figure 3E, *P* adjusted < 0.05; see Methods and Supplemental Data Set 1).

Cell deconvolution analysis of glomerular ROI displayed high abundance of glomerulus-specific cell types (such as podocytes and GECs) and immune cells, but no cells specific to other regions of the nephron (Figure 3F). No major deviations in cell abundances were detected between diseased and nondiseased glomeruli. Deconvolution analysis of the tubule ROI similarly showed high abundance for tubule-specific cell types, but no glomerular cells (Figure 3G). Since our main interest is glomerular damage, we focused our subsequent analysis specifically on glomeruli.

### Pattern of gene expression in AS, FSGS, and MN glomeruli.

We first analyzed all patients with CKD (young and adult patients with AS as well as patients with FSGS and MN) by PCA, which partitioned the glomeruli into distinct disease-specific clusters, except for the patient with PLA2R^+^ MN (no. 7), whose glomeruli clustered with the nondiseased glomeruli (Supplemental Figure 3). Of note, despite the low pathology scores of the PLA2R^+^ glomeruli, the similarity in gene expression between these glomeruli and glomeruli of the nondiseased patients was unexpected (see Discussion for more information).

**Figure 4:**
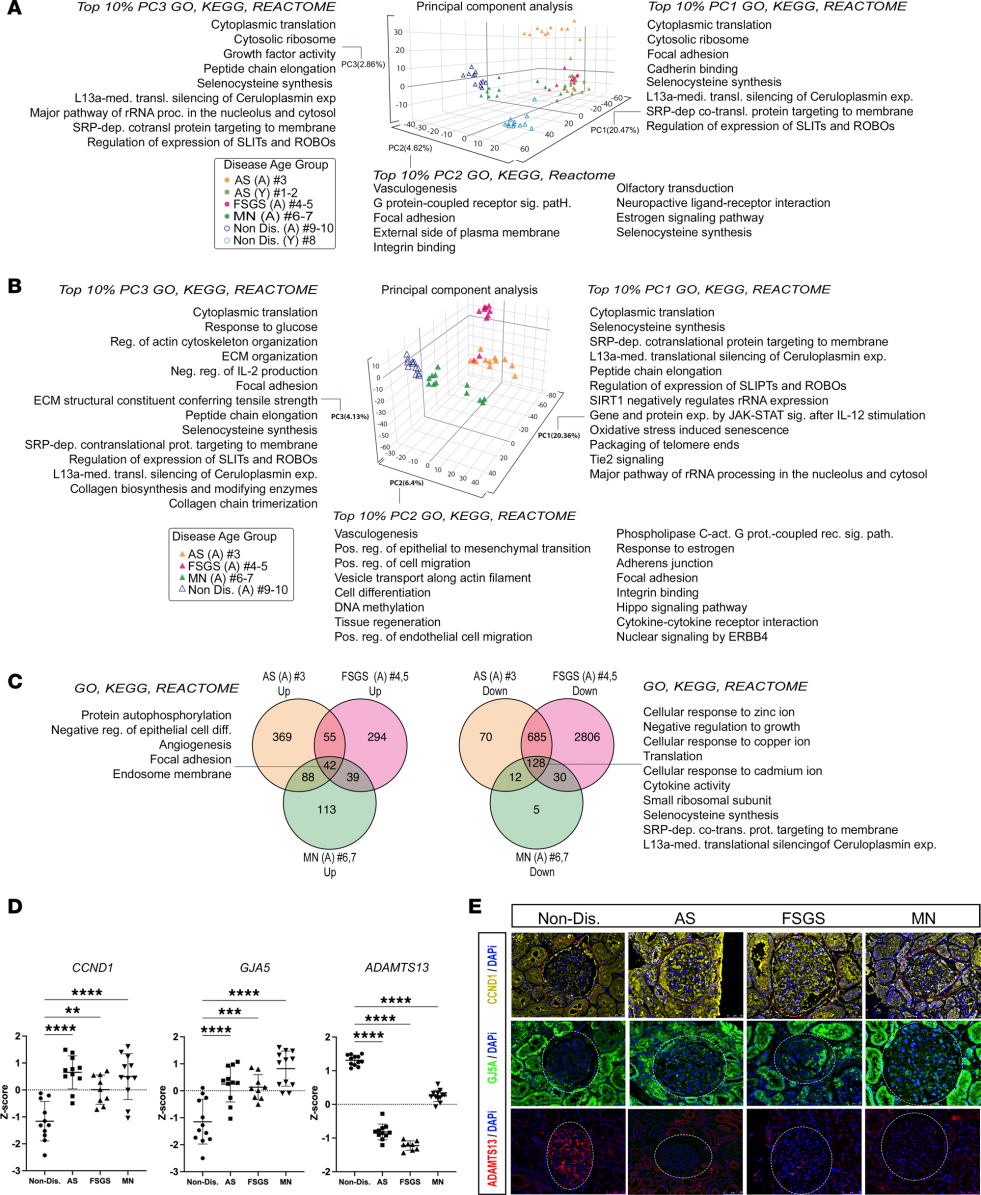
Comparison of transcriptional programs among AS, FSGS, and MN glomeruli. (**A**) Unsupervised principal component analysis (PCA) based on label-free quantification of the transcripts expressed in all glomeruli, based on PC1, PC2, and PC3. Percentage of total variance is indicated on each PC axis. Significantly enriched GO terms and KEGG and REACTOME pathways (EASE-modified Fisher’s exact *P* < 0.05) are listed for the top 10% of transcripts contributing the most to each principal component. (**B**) Unsupervised PCA based on label-free quantification of the transcripts expressed in adult glomeruli, based on principal components (PC1, PC2, PC3). Percentage of total variance is indicated after each principal component. A list of significantly enriched GO terms and KEGG and REACTOME pathways (EASE-modified Fisher’s exact *P* < 0.05) is provided for the top 10% of transcripts contributing the most to each principal component. (**C**) Venn diagrams showing the total number of differentially upregulated and downregulated genes (Student’s *t* test and binomial GLM test BH-adjusted *P* < 0.05) in AS (no. 3), FSGS (no. 4 and 5), and MN (no. 6 and 7) glomeruli as well as nondiseased glomeruli (no. 9 and10). Significantly enriched GO terms and KEGG and REACTOME pathways (EASE-modified Fisher’s exact *P* < 0.05) for the genes commonly upregulated (*n* = 42 genes) and commonly downregulated (*n* = 128 genes) in all the samples are depicted next to the Venn diagrams. (**D**) Box plots depicting the *Z*-scores for *CCND1*, *GJA5*, and *ADAMTS13*, commonly upregulated or downregulated, in all diseased glomeruli versus nondiseased (no. 9 and 10) control glomeruli (Student’s *t* test). **P < 0.01; ***P < 0.001; ****P < 0.0001. Data are shown as the mean ± SD. (**E**) Representative immunofluorescence images of CCND1 (yellow), GJA5 (green), and ADAMTS13 (red) on kidney sections derived from AS, FSGS, and MN and nondiseased kidney tissue. Nuclei are stained blue with DAPI. Dotted lines indicate the glomerular ROIs to distinguish staining in the glomerulus versus tubules. Scale bars: 50 mm.

The top 10% of genes driving the PC1 and PC3 separations included genes associated with cellular translation, selenocysteine synthesis, L13a-mediated translational silencing of ceruloplasmin expression, and SRP-dependent cotranslational protein targeting to membrane. Along the PC2 axis, the separation was driven by genes most highly associated with vasculogenesis, G protein–coupled receptor signaling pathway, integrin binding, and estrogen signaling pathways (Figure 4A and Supplemental Data Set 2).

To find potential commonalities among the different CKDs, we next analyzed only the adult patients with CKD to minimize the effect of age. PCA analysis partitioned the glomeruli of each disease into specific clusters, with the glomeruli from the patient with FSGS having the greatest separation from the nondiseased glomeruli along the PC1 axis (20.36%), followed by AS no. 3 and MN no. 6 (Figure 4B).

The top 10% of genes and associated pathways driving the PC1, PC2, and PC3 separation remained largely unchanged when compared with the PCA, including all the glomeruli shown in Figure 4A and Supplemental Data Set 3. To identify potential genes and/or pathways commonly regulated between all the patients with CKD, we compared their differential gene expression profiles (Student’s *t* test and binomial test). As shown in Figure 4C, we identified 42 genes that were commonly upregulated in all diseased glomeruli, with *TNS1*, *CCND1*, and *GJA5* being the most strongly upregulated genes, and 128 genes that were downregulated with *ADAMTS13* and *HOXB8* being the most downregulated ones (Supplemental Figure 4, A and B, and Supplemental Data Set 4). Changes in these genes’ expression in the glomerular region by DSP (Figure 4D) was also confirmed immunohistochemically (Figure 4E).

We next evaluated whether some of the top DE genes with the highest levels of expression identified in all the glomeruli using DSP were also represented in patients from the NEPTUNE CKD cohort (FSGS, MN, and minimal change disease [MCD]; Supplemental Figure 5A). We confirmed that *GJA5*, *TNS1*, and *CCND1* were also significantly upregulated in the NEPTUNE cohort (Supplemental Figure 5B).

We also analyzed and compared AS and FSGS biopsies independently of MN biopsies (Supplemental Figure 6, A and B, and Supplemental Data Set 5), biopsies from adults with AS against those from young individuals with AS (Supplemental Figure 6, C and D), and each adult disease separately (Supplemental Figures 7–9), the results of which are discussed in the Supplemental Results.

### Associations between histopathology and transcriptional signature in AS, FSGS, and MN glomeruli.

To construct a possible whole-glomerulus progression “pseudotime” representation of gene expression changes, indicating increasing degrees of glomerular injury, we performed a trajectory analysis of diseased and nondiseased glomeruli. This is analogous to the developmental pseudotime trajectory analysis that can be conducted on scRNA-Seq data, except for the transcriptional distances being calculated on a whole-glomerulus rather than cellular level.

**Figure 5:**
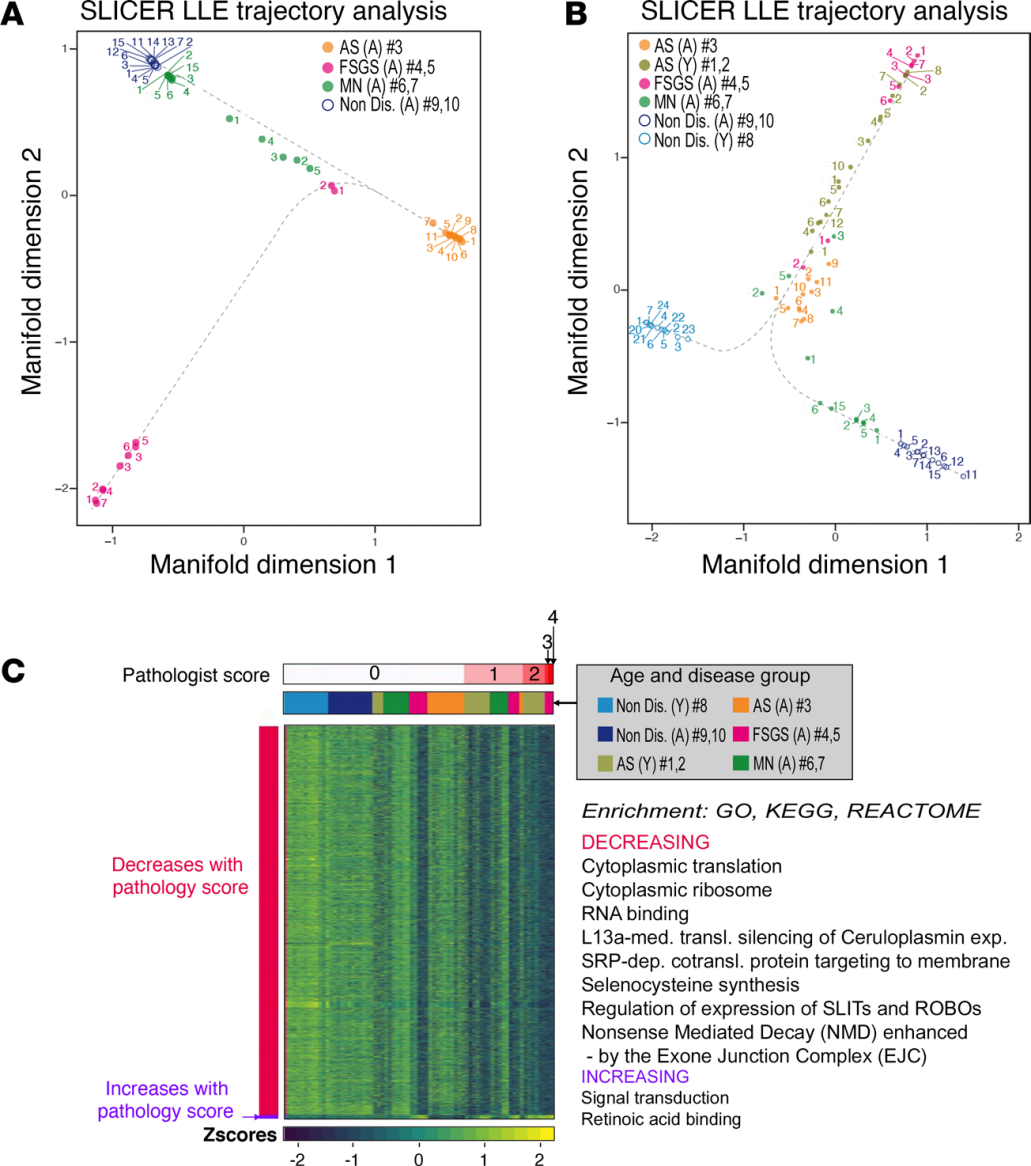
Regression and trajectory analysis. (**A** and **B**) Mapping of gene expression perturbation data to the inferred trajectories by SLICER from a combined analysis of adult AS (no. 3, orange), FSGS (no. 4 and 5, pink), and MN (no. 6 and 7, green) glomeruli and their corresponding nondiseased glomeruli (no. 9 and 10; **A**), and combined analysis of adult AS (no. 3, orange), young AS (no. 1 and 2, gray-green), FSGS (no. 4 and 5, pink), and MN (no. 6 and 7, green) glomeruli and their corresponding nondiseased glomeruli (no. 8, 9, and 10; **B**). The numbers of the glomerular ROIs are depicted next to each data point. The dotted lines represent fitting curves that indicate the relationship between different glomeruli on the trajectory path. (**C**) Pathology scores as an index of progression regression analysis were applied to identify genes and pathways most highly associated with pathological changes in glomeruli across all the biopsies (no. 1–10). The heatmap shows the linear increasing or decreasing trend (likelihood trend fitting *P* < 0.05). Significantly enriched GO terms and KEGG and REACTOME pathways (EASE-modified Fisher’s exact *P* < 0.05) are depicted on the right of the heatmap. A, adult (sample ID no. 3–7 and no. 9 and 10); Y, young (sample ID no. 1, 2, and 8).

When all adult glomeruli were combined into a single trajectory, two separate paths emerged. Compared with the nondiseased glomeruli, the AS (no. 3) glomeruli diverged with respect to the first and second manifold dimensions, while the FSGS (no. 4 and 5) glomeruli diverged only with respect to second manifold dimension, with little to no variation with respect to the first dimension, while MN no. 6 presented a trajectory path trending between AS no. 3 and the nondiseased glomeruli (Figure 5A). The nondiseased glomeruli and MN no. 7 were positioned in a very narrow range with respect to both dimensions; thus, in the overall combined trajectory analysis, the 2 manifold dimensions appear to both represent pathological dimensions, suggesting that the pathological processes driving progression in the disease subtypes diverge (Figure 5A). In addition, when the young glomeruli were combined with the adult glomeruli, the trajectory diverged with respect to the first manifold dimension separating the normal controls of the young and adult patients, suggesting that this dimension represents at least in part developmental processes. In contrast, all the diseased glomeruli (with the exception of MN no. 7) combined into a single trajectory path (Figure 5B).

We also analyzed each disease separately (Supplemental Figure 10). We recognized that the sample size for each individual disease is limited; nevertheless, these data are a proof of principle of the use trajectory analysis in the context of glomerular data for specific diseases. Each case, regardless of disease type or age, resolved into a single trajectory without branching (Supplemental Figure 10, A–D), suggesting that on a whole-glomerulus-level transcriptional changes can be seen as following a “uniform transcriptional path” as the disease progresses over time. Nondiseased glomeruli (both young and adult) were highly constant with respect to the second manifold dimension (except in MN, where a slight variation was noticed in a seeming overlap and continuation of the normal glomerulus trajectory, Supplemental Figure 10D), suggesting that this axis represents the pathological processes in AS (Supplemental Figure 10, A and B) and FSGS (Supplemental Figure 10C), since the nondiseased glomeruli did not participate on this dimension, while the first dimension, over which nondiseased glomeruli showed substantial variation, likely represents normal glomerular physiological variation. The diseased glomeruli clearly varied in both dimensions. Our analysis revealed that some diseased glomeruli (such as glomeruli no. 4, 6, and 7 in patient with AS no. 2; glomeruli no. 2, 3, and 4 in patient with FSGS no. 5, and glomeruli no. 4 in patient with MN no. 6; Supplemental Figure 10, A–D) with greater distances from the nondiseased glomeruli along the trajectory path (thus considered trending toward progressive damage) had a pathological score of 0. This observation suggests that histopathological assessment might not be able to clearly distinguish between transcriptionally normal and/or mildly damaged glomeruli.

Next, despite the significant transcriptional variation evident among glomeruli with pathology scores of 0, we used pathology scores over the entire range as an alternative index of progression. We then performed linear regression analysis of the pathology scores and transcript abundances to identify genes and signaling pathways that correlated the most with these scores.

When analyzed together, glomeruli from patients with AS, FSGS, and MN showed a decreasing trend of expression of genes highly enriched for SRP-dependent cotranslational protein targeting to membrane, selenocysteine synthesis, and regulation of expression of SLITs and ROBOs, among others (Figure 5C and Supplemental Data Set 6).

### Gene correlation analysis identified patterns of gene expression specific to glomerular cell types.

Since interpretation of spatial transcriptomics at the whole-glomerulus level is limited by the fact that transcript quantity is a function of both the level of net transcription (as with single-cell methods) and the number of the cells producing the transcript in the glomerulus, we reasoned that gene-specific transcripts that correlated strongly with several presumed constitutive glomerular cell–type markers would more likely reflect changes in the level of transcription rather than cell number. Therefore, we selected a set of genes recognized to be specific to glomerular cells: *WT1*, *NPHS1*, and *NPHS2* for podocytes; *EHD3*, *TEK*, and *CDH5* for GECs; *PDGFRβ*, *CD44*, and *GATA3* for mesangial cells (22); then we performed correlation analysis (using a *r* +0.5 and *r* –0.5 as a cut-off for meaningful correlation or anticorrelation for all the biopsies combined, young and adult) to detect cell type–specific signatures of gene changes. A complete list of all correlated and anticorrelated genes can be found in Supplemental Data Set 7. We identified 4 transcripts, *PODXL*, *CLIC5*, *HTRA1*, and *TGFBR3*, that correlated with the 3 podocyte markers (Table 2). While *PODXL* is known to be another specific podocyte marker, we believe that its correlation with *NPHS2* in multiple glomerular diseases is new. Even if S100A6 is expressed in other cell types (23), we also determined that it correlated very highly and very specifically with *NPHS2* only. We identified 3 transcripts, *IGFBP5*, *SLC9A3R2*, and *ENG* that correlated with all 3 of the GEC markers (Table 3). We did not find any transcripts that would correlate with all 3 of the mesangial markers, *PDGFRB*, *CD44*, and *GATA3*, but we identified *PTPN12* and *CCND1* as correlating with *PDGFRB* and *GATA3* (Table 4).

### Gene expression and transcriptional programs in young AS glomeruli.

**Figure 6:**
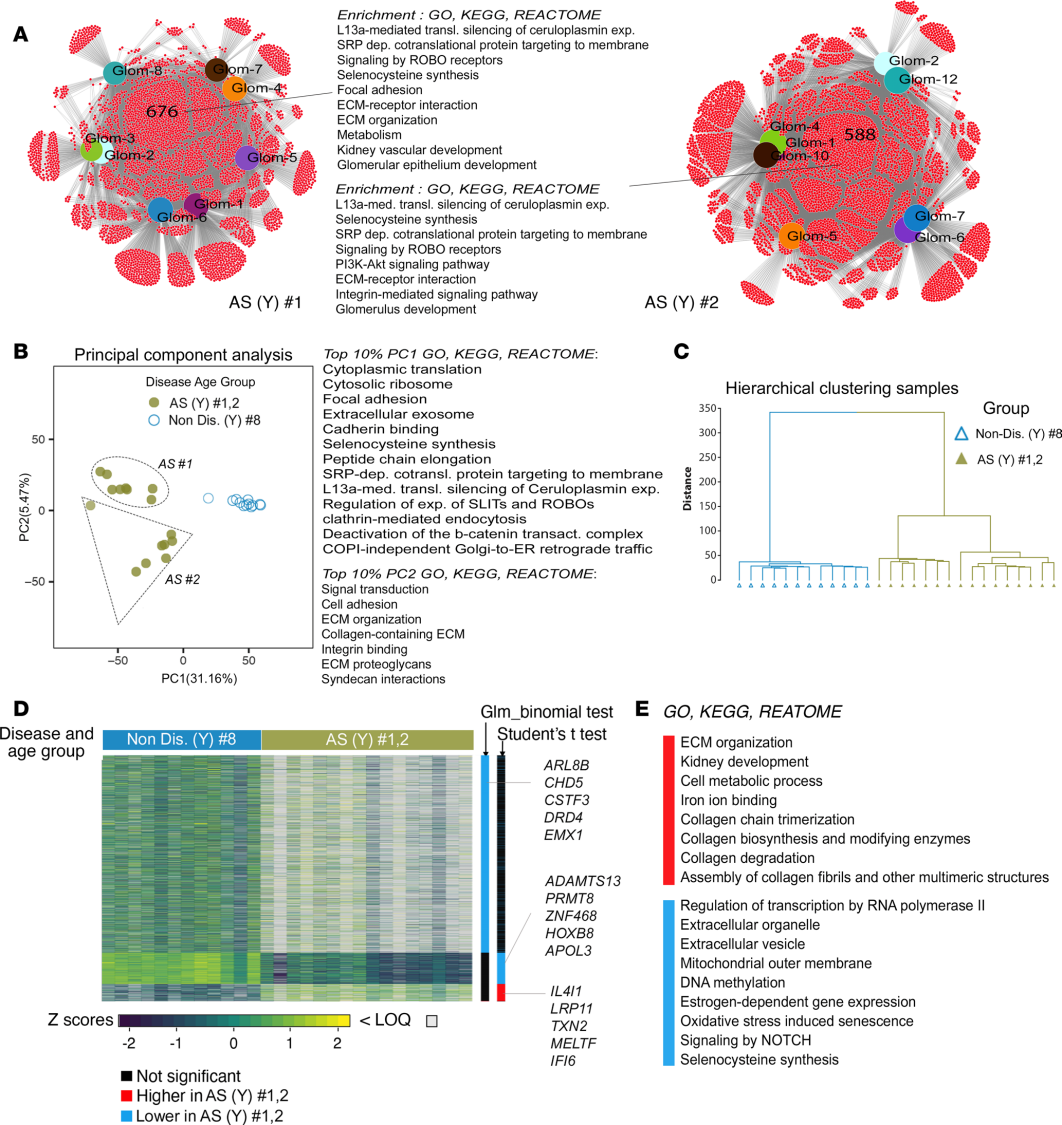
Gene expression signature in AS glomeruli from young patients. (**A**) Venn diagrams of TMM-normalized expression of genes detected above LOQ in biopsies from young patients with AS (sample no. 1, *n* = 8 ROIs; sample no. 2, *n* = 7 ROIs), showing the distribution of the number of genes commonly expressed in all glomeruli (AS no. 1, 676 genes; AS no. 2, 588 genes). The black lines point to a list of the most highly enriched pathways (GO, KEGG, REACTOME) for the commonly expressed genes among all glomeruli in AS no. 1 and 2. (**B**) Unsupervised principal component analysis (PCA) based on label-free quantification of the transcripts expressed in young AS (no. 1 and 2, gray-green circles) and young nondiseased glomerular ROIs (no. 8, blue circles), based on PC1 and PC2 constructed to capture the most variation in the samples. Percentage of total variance is indicated after each principal component. Significantly enriched GO terms and KEGG and REACTOME pathways (EASE-modified Fisher’s exact *P* < 0.05) for the top 10% of transcripts contributing the most to each principal component are shown next to the plot. (**C**) Dendrogram showing hierarchical clustering and transcriptional link between glomeruli from AS no. 1 and 2 and nondiseased glomeruli no. 8. Y, young. (**D**) Heatmap depicting the transcripts significantly modulated between glomeruli from AS (no.1 and 2) and nondiseased glomeruli no. 8 (Student’s *t* test and binomial GLM test, adjusted *P* < 0.05). Transcripts less than LOQ in value are shown in white. (**E**) A select list of GO terms and KEGG and REACTOME pathways significantly enriched (EASE-modified Fisher exact, *P* < 0.05) for the upregulated and downregulated genes in AS (no. 1 and 2) versus nondiseased (no. 8) shown in **D**.

Taking into account the age differences among patients, we decided to investigate the gene expression in the glomeruli (*n* = 16) from 2 young patients with AS (no. 1 and 2) separately from that of the adults. The 8 glomeruli in the AS patient no. 1 shared 676 genes in common. The 8 glomeruli in AS patient no. 2 shared 588 genes in common. These genes were enriched for pathways related to regulation of ceruloplasmin, selenocysteine expression, SRP-dependent cotranslational protein targeting to membrane, and SLITs/ROBOs (Figure 6A and Supplemental Data Set 8).

When glomeruli within the same biopsy were compared, we identified specific “individual” transcriptomic signatures for each of the glomeruli. These glomeruli expressed unique clusters of genes and enrichment patterns, and the number of these genes varied across different glomeruli. The molecular signature for each glomerulus can be found in Supplemental Data Set 8.

To reveal the transcriptional signature of AS glomeruli, we compared their expression data against that of age-matched controls (*n* = 12 glomeruli, biopsy no. 8; Figure 1A) derived from a nondiseased section of a resected kidney from a 6-year-old patient. PCA (Figure 6B) as well as hierarchical clustering analysis (Figure 6C) separated AS glomeruli and nondiseased glomeruli cleanly. Enrichment analysis performed on the top 10% of genes contributing to PC1 and PC2 revealed that the biological functions driving the separation between the AS and nondiseased glomeruli included cytoplasmic translation, focal adhesion, selenocysteine synthesis, and SRP-dependent protein trafficking in PC1 and organization and interactions of ECM in PC2 that separated AS no. 2 moderately from the nondiseased glomeruli. No significant separation of AS no. 1 from the nondiseased glomeruli was present (Figure 6B and Supplemental Data Set 9).

Using binomial test and Student’s *t* test, we identified that the expression of 3,205 genes was altered (adjusted *P* < 0.05) in the glomeruli based on their origin (AS no. 1 and 2 and nondiseased, Figure 6D). Among the top genes commonly overexpressed were *IL4I1*, *LRP11*, *MELTF*, and *TXN2*; and underexpressed genes included *ADAMTS13*, *PRMT8,* *ZNF468*, *HOXB8*, and *APOL3* (Figure 6D). Of note, none of the overexpressed transcripts in the young AS no. 1 and 2 were found to be differentially regulated in the adult AS no. 3 glomeruli; however, the same underexpressed transcripts were downregulated also in the adult AS no. 3 glomeruli. Some of these differences may be due to age differences between the young and adult samples or alternatively based on the different nondiseased kidney used as reference. Enrichment analysis of DE genes revealed that genes upregulated in the AS glomeruli were enriched for GO terms associated with ECM organization, collagen biosynthesis and degradation, and cell adhesion. The downregulated genes were enriched for GO terms associated with regulation of cellular transcription, DNA methylation, signaling by NOTCH, and selencysteine synthesis (Figure 6E and Supplemental Data Set 10).

## Discussion

Transcriptomic technologies, including bulk RNA-Seq, scRNA-Seq (24, 25), and snRNA-Seq methods (26), have allowed in-depth characterization of kidney cell subtypes and their biological roles in health and disease (27), but they cannot connect the transcriptional data to its spatial location within the kidney (28). This is particularly important in the context of kidney disease, in which different regions of the kidney can show significant heterogeneity in the extent of injury. Disease heterogeneity can occur across various glomeruli or tubules due to differences in progression of the basic mechanisms or to local effects of fibrosis, such as glomeruli near fibrotic tubules that may be affected by inflammatory tubule products, independent of their own injuries.

Here, using a variety of computational methods, and after establishment of stringent quality control parameters, we present the application of DSP technology on the GeoMx platform to study the spatial glomerular transcriptional signatures in kidney biopsies from patients with AS, FSGS, and MN. We decided to focus on the glomerulus because damage to this structure is one of the major causes of progressive CKD that often leads to kidney failure (1, 2, 29). Our goal was to establish a proof of principle that DSP could provide valuable spatial information on glomerular gene expression across different types of glomerular diseases. With the use of this technology, we also aimed to elucidate a transcriptional signature that may identify common pathways of progression of the diseases we studied.

We have previously shown that the DSP approach can accurately detect glomerulus-specific and tubule-specific gene expression signature in a kidney graft biopsy from a patient with a chronic/active T cell–mediated rejection episode and nondiseased kidney control biopsies (30). In the current study, in addition to using tubule regions as an internal control, we performed cell deconvolution analysis, which further confirmed that our selection method (identification of glomerular or tubular ROI) accurately detects nephron segment-specific transcriptional profiles, thus allowing us to compare the transcriptional data of a single glomerulus to its histopathology and to study interglomerular (in the same biopsies) or interindividual (between different biopsies) heterogeneity in glomerular gene expression.

We acknowledge that we did not sequence glomeruli that had very few cells, as these were much smaller in size and, thus, were beyond the level of sensitivity of the DSP assay; these glomeruli also tend to be mostly acellular, with high fibrosis, and would therefore probably be transcriptionally uninformative. Therefore, based on our selection criteria, the selected AS, FSGS, and MN glomeruli that we studied probably predominantly represent the earlier-to-intermediate stages of disease progression. Here, we used a limited number of biopsies per disease category owing to limited availability of samples. Nevertheless, we analyzed 73 glomeruli in total, which can be considered enough biological replicates as a pilot analysis using this technology applied to these specific glomerular diseases. We also recognize that control glomeruli may have undergone transcriptional changes due to nonpathological processes. However, evidence that the glomeruli we selected in the age-matched nondiseased tissue that was used as a control had low variability of gene expression increases our confidence in our data interpretation.

An ability of the DSP technology to quantitate transcript levels in defined glomerular regions is a powerful approach to studying disease heterogeneity. Our analysis identified specific genes and pathways that were enriched in the glomeruli of each patient (Supplemental Results). However, these individual results cannot be generalized due to the limited sample size for each disease; therefore, we found it more informative to pool all the samples together to highlight findings that were common among the glomerular diseases. All the glomeruli except those from PLA2R^+^ MN patient no. 7, clustered differently from the nondiseased glomeruli and showed alterations of common pathways that are known to be altered in AS (ECM remodeling, integrin mediated signaling; refs. 12, 31–34), in FSGS (ECM organization, TGF-β receptor signaling; refs. 35, 36), and in MN (inflammatory response and MAPK pathway, ref. 37). The PLA2R^+^ MN showed a transcriptional signature very similar to that of the nondiseased glomeruli. We have no clear explanation for why this is the case, although it is possible that the causative effect of an autoantibody leads to a more limited mechanistic injury to only a single cell type in the glomerulus or possibly even small changes at the transcriptional level that we detected (Supplemental Figure 9C) might still be able to produce a biological effect on the development of the disease. For example, despite the limited differences between PLA2R^+^ MN and nondiseased control glomeruli, we still found significant transcriptional regulation of *CCND1*, *GJA5*, and *ADAMTS13* (Supplemental Figure 11 and Supplemental Data Set 16). We also found enrichment in biological processes and pathways associated with vasculogenesis and integrin-mediated signaling (Supplemental Data Set 16).

Our DSP analysis identified a transcriptional signature driving the separation between the nondiseased controls and all the biopsies from patients with AS (young and adult), FSGS, and MN, independently of their age, etiology, or pathological scores. One of them, SRP-dependent cotranslational protein targeting to membrane signaling, which plays an important regulatory role in protein-folding homeostasis (38), was consistently downregulated in all diseased glomeruli. Accumulation of misfolded proteins in the endoplasmic reticulum (ER), which leads ER stress, has been described in experimental models of proteinuric kidney disease as well as in human kidney biopsies, including AS (39, 40), FSGS, and MN (41). In podocytes and mesangial cells, ER stress can be induced by various factors, including oxidative stress, changes in cellular lipid concentrations, or genetic mutations (42, 43). In response, the ER activates quality control mechanisms, including the unfolded protein response pathway, to counterbalance the effects of ER stress (44). Therefore, consistent with previous reports, our data suggest that protein misfolding and ER stress might be important common progression factors in different glomerulopathies. Another common pathway we found, also downregulated in all glomeruli, was associated with selenium metabolism. This is consistent with recent reports demonstrating that patients with acute kidney injury or CKD are frequently found to have low serum selenium levels (45). Selenium is known to influence antioxidant mechanisms (46, 47), thus suggesting that its deficiency could potentially impede the kidney’s ability to effectively cope with oxidative stress. We also identified transcriptional programs related to angiogenesis and focal adhesion to be upregulated in AS, FSGS, and MN. This is consistent with findings from many reports demonstrating the involvement of an imbalance of angiogenesis-linked mediators (such as VEGF-A) in the progression of CKD (48, 49). Focal adhesions, involving integrins, play an important role in podocyte foot process structure, and numerous studies (50, 51) have suggested that targeting these dynamic structures has the potential to improve treatments for CKD.

DSP identified 128 specific downregulated genes and 42 specific upregulated genes common to glomeruli from all the adult diseased biopsies, 87 of which were also DE (adjusted *P* < 0.05) in the young AS glomeruli (expression in all diseases was also validated histochemically). Importantly, we confirmed that 3 genes *GJA5*, *TNS1*, and *CCND1* were also significantly upregulated in the NEPTUNE cohort (Supplemental Figure 5). For example, it is known that CCND1 plays an important role in cell cycle entry (G_1_/S transition) and cell proliferation/migration. It has been reported that CCND1 is upregulated in podocytes in idiopathic collapsing glomerulopathy (52), and in other glomerular cells and diseases (52, 53), but additional experiments will be required to identify the specific cell type responsible for this upregulation. Even if the role of *GJA5*, *TNS1*, and *CCND1* in glomerular disease requires further investigation (beyond the scope of this work), they might represent a biomarker signature of progressive glomerular damage, independent of the etiology. Of note, even if not all the genes were significantly upregulated or downregulated, the directionality of gene regulation was retained across most genes that we have investigated. This difference is not surprising, given that the references used in these analyses like biopsies from partial nephrectomies and biopsies from living donors might be different; here, we did not have access to biopsies from living donors, and our patient cohort had differences in disease etiology; for example, we did not investigate MCD, and AS data were not available in the NEPTUNE Cohort. Nevertheless, we think that identification of 3 common upregulated genes and a trend in DE are sufficient to support validation of our method.

Furthermore, using trajectory analysis, we showed that glomeruli can be placed along whole-glomerulus pseudotime trajectories. Analogous to developmental pseudotime, as often represented in scRNA-Seq analyses (54), the location of the nondiseased glomeruli in the trajectory plot suggests that the diseased glomeruli may represent different points in a pathological progression timeline. It is of interest that our glomerular histology assessment did not completely capture the heterogeneity of diseased glomeruli, with most of the glomeruli looking histologically unremarkable, despite showing clear signs of a transcriptional trajectory, thus allowing us to conclude that histological evaluation may not be sufficient to evaluate pathological events occurring at the transcriptional level. It is possible that trajectory analysis of relatively uninjured glomeruli can give insight into the earliest aspects of pathologically relevant changes that will eventuate in irreversible structural damage, perhaps at a stage when appropriate therapies could return the glomeruli to normal physiology.

In addition, our gene correlation analysis showed how gene expression programs in podocytes and glomerular endothelial and mesangial cells relate to one another in these diseases. It was unexpected to find that little to no correlation was present among the major podocyte markers *WT1*, *NPHS1*, and *NPHS2*. However, all of these podocyte markers consistently correlated with *PODXL*. In addition, our analysis found strong correlation between *NPHS2* and *S100A6* (*r* = 0.9134), suggesting that *S100A6* maybe a good candidate as an alternative marker for assessing podocyte loss and damage in diverse glomerular pathologies. Additionally, correlation data between *GJA5* (one of the common signature genes upregulated in our biopsies and in the NEPTUNE Cohort) and *EHD3* (GEC-specific marker) and our immunohistochemistry data suggest *GJA5* as a potential new marker for GEC.

Overall, our data have shown that, despite of its limitations regarding sequencing depth, DSP is a very useful platform for generating transcriptional maps of kidney glomerular and tubule structures. With good sample preparation and strong analytical approaches, DSP can be a powerful tool to study compartment specific transcriptional programs in the kidney. Indeed, our analysis showed that a whole-glomerulus analog to pseudotime development trajectories in single cells exists that seems to describe an analogous pathological trajectory on the glomerular level. Further studies of these networks are warranted to uncover pathways or genes that might be targeted at early disease stages to prevent progression in a variety of glomerular disorders.

## Methods

### Sex as a biological variable.

Our study examined kidney biopsies from male (*n* = 6) and female (*n* = 4) individuals with AS, FSGS, and MN and individuals without disease acting as controls. All samples were pooled together for analysis, and sex was not considered as a biological variable.

### Biopsy procurement.

For the NanoString GeoMx DSP analysis and for the fluorescence in situ hybridization, paraffinized kidney biopsy samples from individuals with AS, FSGS, and MN and individuals without disease acting as controls were obtained from the pathology biorepository at Mount Sinai Hospital, New York, New York, USA, and IRCCS Istituto Giannina Gaslini. Patient information is reported in Table 1.

Additional AS biopsy specimens used for histology were obtained from University of Utah Health-Pathology Department, and archived biopsy samples were included in this study. These biopsies were previously procured for medical reasons and not as part of a study.

### Histopathology.

Serially cut 3 mm thick sections of formalin-fixed and paraffin-embedded kidney samples from patients with AS, FSGS, and MN (and nondiseased kidney samples) were processed for H&E staining to assess kidney morphology. Briefly, slides were deparaffinized in Citrisolv, and H&E staining was performed using Selectech reagent system on a Leica BOND-RX. Images were captured on a Zeiss Axio Scan Z1 slide imager. Slides were evaluated for glomerular pathology scores by a kidney pathologist. 24 nondiseased glomeruli were used as a reference. For the pathological scoring, a glomerular injury score was given to each glomerular cross-section, based on 7 criteria: glomerular size area, GBM thickness, mesangial expansion, mesangial hypercellularity, intracapillary hypercellularity, FSGS, and global glomerular sclerosis. Each criterion was assigned 1 for the presence and/or change of the criterion or 0 for its absence/no change; the total possible injury score ranged from 0 to 7.

### Immunostaining for ROI identification.

Serially prepared sections (previously hybridized with WTA probes) were also immunostained with CD3, Ki-67, ɑ-smooth muscle actin, and SYTO83 to help with ROI selection. Briefly, Morphology Marker Solution was prepared in the following proportions per slide: 22 μL SYTO (Thermo Fisher Scientific, catalog S11364), 5.5 μL ɑ-Smooth Muscle Actin (NanoString Technologies), 5.5 μL CD3 (NanoString Technologies), 5.5 μL Ki-67 (NanoString Technologies), and 181.5 μL Buffer W for a total volume of 220 μL/slide. Slides were incubated in this solution at room temperature for 1 hour followed by 2 successive SSC washes, after which, the slides were loaded into the NanoString GeoMx Digital Spatial Profiler, where overview scans were captured at ×20 to guide the selection of ROIs.

### Glomerular ROI selection criteria.

Glomerular ROIs were arbitrarily selected in all biopsies, guided by the morphology markers as described above. In the diseased biopsies, glomeruli that lacked enough cell numbers and, therefore, genetic material for quality data output, could not be selected for downstream DSP analysis. In the nondiseased control samples, we arbitrarily selected those glomeruli that histologically appeared unremarkable and were not adjacent to any tubular or interstitial lesions.

### Sample processing for NanoString GeoMx DSP and quality control analysis.

Slides were prepared for hybridization following a modified RNA FFPE BOND RX Slide Preparation Protocol in the GeoMx NGS Slide Preparation User Manual (NanoString Technologies, MAN-10115). We acknowledge that biopsy tissue was not available for RIN or DV200 analysis, and this represents a limitation in our study design. Briefly, deparaffinization, antigen retrieval, and proteinase K digestion were performed on a Leica BOND-RX instrument with Bond Dewax Solution, Bond Epitope Retrieval Solution 2, and 1 mg/mL proteinase K digest at 37°C. Human WTA probes (NanoString Technologies, catalog 121401102) were hybridized at 4 nM each in Buffer R (Nanostring Technologies) and incubated in a hybridization chamber for 16 hours at 37°C. Two washes in 50% Formamide/2x SSC (Thermo Fisher, catalog AM9342/MilliporeSigma, catalog S6639) at 37°C washed nonspecifically bound probes from the slides, which were then blocked prior to antibody staining with 200 μL Buffer W (Nanostring).

After the staining, DSP operators defined individual tubules and glomeruli in the DSP software as individual geometric segments, with sizes ranging from 9,700 to 168,000 μL, guided by markers and H&E staining (Leica Biosystems, catalog 3801571 and 3801616) (see *Glomerular ROI selection criteria*). The WTA probe tags were selectively UV-cleaved and gathered from each of these ROIs and transferred to individual wells of a collection plate on the DSP. Library preparation was performed according to the GeoMx-NGS Readout Library Prep User Manual (NanoString Technologies, MAN-10117). The collection plates were dried down and resuspended in 10 μL nuclease-free water to ensure uniform volume. 4 μL of this tag suspension was added to a PCR plate and amplified as per the protocol. PCR products were pooled and purified by 2 rounds of AMPure XP beads (Beckman Coulter, catalog A63882), with the quality of the resulting library assessed by a Bioanalyzer DNA High Sensitivity assay (Agilent, catalog 5067-4626). The library was sequenced on an Illumina NextSeq 1000/2000, after which the GeoMx NGS Pipeline v1.0.0 (NanoString) processed fastq files, tabulating raw counts for each gene and ROI.

We used an outlier detection test to eliminate certain ROIs from subsequent analysis. We performed our outlier detection based on a rMd, with a *P* value threshold of 0.0001. This distance was computed using the correlation coefficient; the fraction of the data missing; the median absolute deviation of transcripts, which is a robust measure of the spread of the data; the skewness, which is a measure of data symmetry of the data symmetry; and the kurtosis, which measures “heavy-tailedness.” The calculation of the rMd was performed using the package pMartR v2.0 as previously described (55, 56).

### Data visualization matrices and analysis methodology.

Venn diagrams in Figure 6A; Supplemental Figure 7A; Supplemental Figure 8, A and B; and Supplemental Figure 9, A and B, were realized using “DiVenn” (57). For these select Venn diagrams, the functional enrichment analysis for GO terms, KEGG, and Reactome pathways was performed using g:Profiler (version e105_eg52_p16_e84549f) with g:SCS multiple testing correction method applying significance threshold of 0.05 (58). All primary analysis is reported in Supplemental Data Sets 1–16.

Our data were also compared with the NEPTUNE cohort glomerular transcriptomic signature of patients with FSGS, MN, and MCD and patients with living donors (Supplemental Figure 5A). Briefly, bulk RNA-Seq was performed in microdissected glomeruli, data were normalized, and DE was calculated using Limma and Limma-Voom.

### Deconvolution analysis for glomerular and tubular cell types.

Cell deconvolution was performed using the SpatialDecon package in R (NanoString, https://github.com/Nanostring-Biostats/SpatialDecon.git; commit ID 94fe087) with sample-wise counts and the Adult Kidney_HCA cell profile (https://github.com/Nanostring-Biostats/CellProfileLibrary.git; commit ID 6b0fd2c) as inputs. Publicly available scRNA-Seq experiments were compiled into profile matrices by aggregating gene-wise counts of all annotated cell types. The spatialdecon function estimated abundances of each cell type within each ROI by comparing its gene expression to each cell-specific gene expression pattern of the profile matrix.

### Immunohistochemistry.

Thin deparaffinized kidney sections (5 μm), representative of AS, FSGS, and MN and corresponding nondiseased controls, were deparaffinized followed by antigen retrieval. Slides were subsequently blocked in 3% BSA and immunostained against GJ5A (Abcam, ab213688), CCND1 (Thermo Fisher Scientific, 595-MSM1-P1ABX), and ADAMTS13 (Thermo Fisher Scientific, PA5-103577) at a concentration of 1 μg/μL, with an overnight incubation at 4°C, followed by AF555-conjugated secondary antibody at a 1:100 dilution; they were mounted with DAPI to visualize nuclei. Images were captured with a Leica DM RA fluorescent microscope in conjunction with Open Lab 3.1.5 software.

### Statistics.

Data processing was performed in R v4.2.1. The RNA count of the negative probes was used to evaluate the LOQ. In brief the median and SD of the negative probe counts were calculated, and we defined the LOQ to be 3 SDs above the median of the negative probe counts. RNA counts below this threshold were converted in missing values. Then, the count data were normalized using the trimmed mean of M values (TMM) method with the package EdgeR (v3.40.2). Statistical analysis and figures were generated using the package RomicsProcessor v1.0.0, which is available on Github (https://github.com/PNNL-Comp-Mass-Spec/RomicsProcessor/commit/72d35c987900febc3e6c6ed416d4d72dc5820075) (59). The large data set was subsided to perform statistics and comparisons among different groups. PCA and hierarchical clustering were performed to verify the grouping of the sample within each subset. Two-tailed heteroscedastic Student’s *t* tests were performed when at least 50% of the samples from each group to be compared were available. To statistically evaluate if the difference between presence and absence of quantitative value between 2 groups was significant, we used binomial GLM tests. For each test performed, the *P* value frequency was plotted to ensure that the *P* value threshold was selected appropriately. The *P* value filter employed was the Benjamini-Hochberg-adjusted *P* < 0.05 for both *t* tests and binomial GLM. One-way ANOVA was applied for multiple comparisons. The linear model of the normalized transcript abundances as the dependent variable was fit to the pathological scores (model equation, *yi* = *β0* + *β1* × *xi* + *ϵi*, where *x* and *y* represent the log-transformed normalized transcript abundance and the log-transformed pathological score [plus 1, to avoid 0 values], respectively; *i* represents the region of interest; *β0* is the ordinate at origin; *β1* is the slope; and *ϵ* the error of estimation). Likelihood ratios tests were conducted to identify the proteins with evidence of significant linear trends with pathological score. Venn diagrams were realized using the package “ggvenn” v0.1.9. Pearson’s correlations were used to identify RNA with abundances positively or negatively correlated to the abundance of transcripts of genes known to participate in glomerular diseases. Trajectory analyses were performed using SLICER v0.2.0 (https://cran.r-project.org/src/contrib/Archive/SLICER/). Enrichment analyses were performed using the homemade package Protein_Minion v0.2.0 (https://github.com/GeremyClair/Protein_MiniOn/commit/76d14a475f5107287ac8b1f09ac37d461a7fa94b) and the EASE score (DAVID’s modified Fisher’s exact tests; ref. 60) on GO terms and KEGG (https://www.genome.jp/kegg/) and Reactome (https://reactome.org) pathways harvested from Uniprot (https://www.uniprot.org, accessed January 30, 2022). The code to perform the analysis was also uploaded on Github to make the data analysis reproducible (https://github.com/GeremyClair/The_spatially_resolved_transcriptome_signatures_of_glomeruli_in_chronic_kidney_disease/commit/75ad58604abd0c59140280f9bc1df98f93431582).

### Study approval.

The institutional review boards for Icahn School of Medicine at Mount Sinai, IRCCS Istituto Giannina Gaslini, and University of Utah Health approved the protocols for the collection of human samples. Archived biopsy samples were included in this study. These biopsies were previously procured for medical reasons and not as part of a study. A waiver of consent was obtained for samples with institutional review board approval.

### Data availability.

The analysis pipeline is available in the Github repository at https://github.com/GeremyClair/The_spatially_resolved_transcriptome_signatures_of_glomeruli_in_chronic_kidney_disease/commit/75ad58604abd0c59140280f9bc1df98f93431582

The authors declare that the main data supporting the findings of this study are available within the article and its supplemental files. Extra data are available from the corresponding author upon request.

In addition, the raw and processed data are also available in the Gene Expression Omnibus repository (GSE255265). Values for all data points in graphs are reported in the Supporting Data Values file.

## Author contributions

GC contributed to analysis and interpretation of the data and revision of the manuscript. HS performed experiments and contributed to analysis of data. PC and AA provided clinical samples and contributed to the revision of the manuscript. FS contributed to the histopathology studies. LAR provided AS human biopsy samples and contributed to the revision of the manuscript. REDF contributed to analysis and interpretation of the data and preparation of the manuscript. SDS contributed to analysis and interpretation of the data and preparation of the manuscript. KVL contributed to the design, analysis and interpretation of the data, and preparation of the manuscript. SS performed experiments; contributed to the design, analysis, and interpretation of the data; and preparation of the manuscript. LP contributed to the design, analysis, and interpretation of the data and preparation of the manuscript.

## Supplementary Material

Supplemental data

Supplemental data set 1

Supplemental data set 2

Supplemental data set 3

Supplemental data set 4

Supplemental data set 5

Supplemental data set 6

Supplemental data set 7

Supplemental data set 8

Supplemental data set 9

Supplemental data set 10

Supplemental data set 11

Supplemental data set 12

Supplemental data set 13

Supplemental data set 14

Supplemental data set 15

Supplemental data set 16

Supporting data values

## Figures and Tables

**Table 4:**
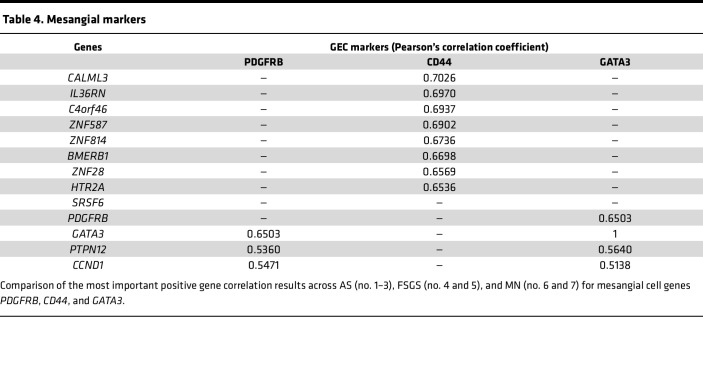
Mesangial markers

**Table 1:**
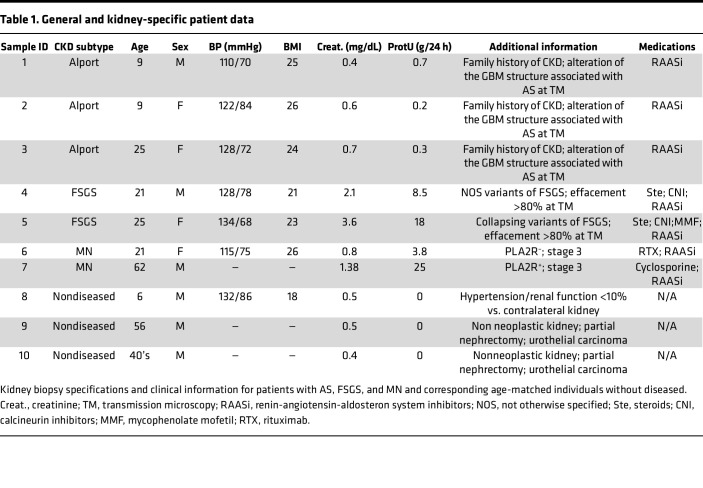
General and kidney-specific patient data

**Table 2:**
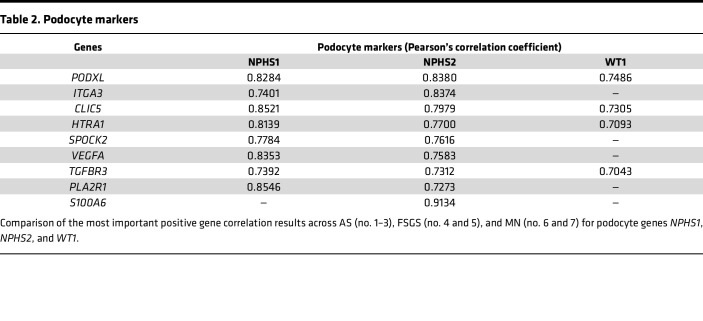
Podocyte markers

**Table 3:**
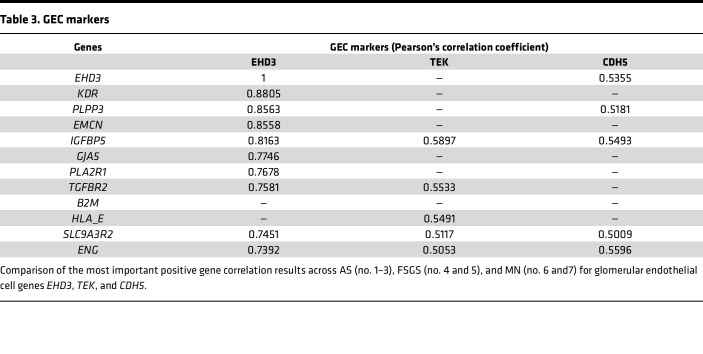
GEC markers

## Version 1 03/22/2024

Electronic publication
