# Supplementary material for: The spatially resolved transcriptome signatures of glomeruli in chronic kidney disease
Source: JCI Insight. 2024 Mar 22;9(6):e165515. doi: 10.1172/jci.insight.165515 (PMC11063942; doi:10.1172/jci.insight.165515)
Supplement: Supplemental data [file jciinsight-9-165515-s152.pdf]

The spatially resolved transcriptome signatures of glomeruli in chronic kidney disease

Supplementary Results

DSP reveals transcriptional programs in adult AS and FSGS glomeruli

Because some cases of familial FSGS present with *ColIV* gene mutations (1-3), and share some common pathological features with AS, we determined if AS and FSGS glomeruli share patterns of gene expression. As shown in the PCA in Figure 4A, major differences between AS #3 and non-diseased glomeruli #9, #10 based on the top 10% of genes in PC2 and PC3 are associated with focal adhesions and G protein-coupled receptor signaling pathway, but little difference in PC1. In contrast, the glomeruli from the FSGS biopsies #4, #5 separated from controls predominantly in PC1, with the top 10% of genes driving the PC1 separation associated with focal adhesion, selenocysteine synthesis and SRP-dependent co-translational protein targeting to membrane (Suppl. Dataset 2).

Our analysis revealed 930 common genes that were differentially expressed (97 upregulated and 833 downregulated) in the glomeruli of AS and FSGS vs non-diseased glomeruli. We performed enrichment analysis on the 97 genes that were commonly upregulated and found pathways related to mitotic cell cycle phase transition, angiogenesis and Hippo signaling (Suppl. Figure 6A). We also performed enrichment analysis on the 833 genes that were commonly downregulated and found pathways associated with G protein-coupled receptor signaling pathway (Suppl. Figure 6B; Suppl. Dataset 5). Complete results of the enrichment analysis of those genes differentially up or downregulated between each disease and non-diseased control, as shown in the Venn diagram in Suppl. Figure 6A and B are reported in Suppl. Dataset 5).

DSP reveals transcriptional programs in young and adult AS

We compared the transcriptional profiles of glomeruli in the young AS to that in the adult AS patients to determine potential similarities and differences. PCA analysis clearly revealed that young and adult AS glomeruli have small but measurable transcriptional differences (Suppl. Figure 6C). Enrichment analysis based on the top 10% of genes in PC1 and PC2 revealed pathways associated with cytoplasmic translation, selenocystein synthesis, and SRP-dependent co-translational protein targeting to membrane among others (Suppl. Dataset 11). Analysis of the genes differentially upregulated in young AS vs adult AS glomeruli showed enrichment in gene ontology terms associated with regulation of apoptosis and transcriptional pathway (Suppl. Figure 6D, Suppl. Dataset 12). Our analysis found no enrichment for the downregulated genes. These results may suggest potential differences in the

glomeruli between young and adult AS patients in the cellular mechanisms related to protein production and processing, such as selenocysteine synthesis (4,5).

DSP reveals transcriptional programs in adult AS glomeruli

We performed DSP on an adult AS patient (#3) separately from the young AS patients because of the age difference and other CKDs to reveal patient specific transcriptional features and used non-diseased glomeruli from two adult patients as a reference control (Table 1).

We analyzed all the detected genes, and transcripts commonly expressed (n=1,058) in all glomeruli (n=11) showed a very similar enrichment pattern to that observed in the young AS patients, including L13a-mediated translational silencing of ceruloplasmin expression, selenocysteine synthesis and ECM-receptor interactions (Suppl. Figure 7A, Suppl. Dataset 13).

We compared the adult AS glomeruli with non-diseased glomeruli from adult patient nephrectomy specimens (n=12, derived from two different patients #9, #10; 6 glomeruli per biopsy. PCA partitioned the AS and non-diseased glomeruli into distinct clusters (Suppl. Figure 7B, Suppl. Dataset 13). GO enrichment analysis performed on the top 10% of genes contributing to PC1 and PC2 revealed that the functions separating the AS glomeruli and non-diseased glomeruli are associated with extracellular matrix, focal adhesions and AGE-RAGE signaling. These pathways were also found in the young AS PCA analysis (Figure 6B), indicating potential similarities.

Transcripts with higher expression in AS #3 relative to non-diseased (#9, #10) glomeruli were enriched in pathways commonly observed in CKD, such as Hippo and VEGF signaling. In addition, pathways associated with focal adhesion, vasculogenesis, kidney development and PI3K-Akt signaling were also highly enriched. Transcripts with lower expression were enriched for RNA polymerase dependent transcriptional regulation, G-protein coupled receptor signaling and pathways not previously associated to AS, such as cellular response to zinc, cadmium and copper ions and as well as olfactory receptor activity (Suppl. Figure 7C, Suppl. Dataset 13).

DSP reveals transcriptional programs in FSGS glomeruli

We investigated the transcriptional changes in FSGS glomeruli following the same analysis scheme as with the AS glomeruli. Genes commonly expressed in all glomeruli (n=1,205 genes in FSGS #4, and n=636 genes in FSGS #5) were enriched in pathways associated with amino acid and protein metabolism including selenocysteine synthesis and L13a-mediated translational silencing of ceruloplasmin expression, cell-ECM interactions, and actin cytoskeleton organization. In addition, glomeruli in FSGS #5 were enriched in gene ontology terms linked to podocyte differentiation and glomerulus development (Suppl. Figure 8A-B; Suppl. Dataset 14).

We next compared the transcriptomes between FSGS and age-matched non-diseased glomeruli. The expression profiles of the two groups clearly separated on the PCA (Suppl. Figure 8C). GO enrichment analysis performed on the top 10% of genes contributing to PC1 revealed that the functions driving the separation were mainly related to selenocysteine synthesis, SRP-dependent co-translational protein targeting to membrane and L13a-mediated translational silencing of Ceruloplasmin expression. Minimal separation was detected in PC2 with functions related to vasculature development and function, cell-ECM interactions and G protein-coupled receptor signaling pathway (Suppl. Figure 8C, Suppl. Dataset 14). It is noticeable that FSGS biopsies displayed greater interglomerular-heterogeneity when compared to the non-diseased controls (Suppl. Figure 8C). Transcripts with higher expression in FSGS glomeruli (both biopsies combined) relative to non-diseased (#9, #10) glomeruli were predominantly enriched in pathways related to ECM remodeling and collagen biosynthesis. Transcripts with lower expression were enriched for expression of olfactory receptors (Suppl. Figure 8D, Suppl. Dataset 14).

DSP reveals transcriptional programs in MN glomeruli

We also analyzed glomeruli affected by MN from a PLA2R-autoantibody-negative (#6) and a PLA2R-autoantibody-positive (#7) patient. PLA2R is defined as the dominant glomerular (podocyte) target epitope for autoantibodies leading to membranous nephropathy (6). Patients negative for anti-PLA2R autoantibodies are more likely to undergo spontaneous remission of nephropathy.

In the biopsy of the PLA2R (+) patient, genes common to all glomeruli (n=2,981) were highly enriched in transcriptional programs related to mitochondrial metabolic processes, vascular development, ECM organization and signaling by NOTCH and WNT pathways. (Suppl. Figure 9A, Suppl. Dataset 15). In the PLA2R (-) patient, genes common to all glomeruli (n=1,010) were highly enriched in transcriptional program similar to that of PLA2R (+) biopsy but also include pathways such as AGE-RAGE and selenocysteine synthesis pathways (Figure 9B, Suppl. Dataset 15).

We compared the transcriptome in the MN glomeruli with age-matched non-diseased glomeruli (n=12 glomeruli, #9, #10): glomeruli from the PLA2R (+) patient #7 clustered together with the non-diseased glomeruli, while the glomeruli from the PLA2R (-) patient #6 clustered separately with greater dispersion along both the PC1 and PC2 axes (Suppl. Figure 9C). GO enrichment analysis performed on the top 10% of genes contributing to PC1 and PC2 revealed that the functions driving the separation between the MN glomeruli in #6 and non-disease glomeruli were related to regulation of integrin mediated cell adhesion and interferon-mediated signaling pathway in PC1, and Rap1, Ras, PI3K-Akt signaling pathways among others in PC2 (Suppl. Figure 9C, Suppl. Dataset 15).

Transcripts with higher expression in MN (#6, #7) relative to non-diseased (#9, #10) glomeruli were enriched in transcriptional program related to VEGF signaling and vasculogenesis. In addition, pathways associated with PI3K-Akt, MAPK, IL17, Hippo and apelin signaling were also highly enriched. Transcripts with lower expression were enriched for selenocysteine synthesis, SRP-dependent cotranslational protein targeting to membrane, L13a-mediated translational silencing of Ceruloplasmin expression and pathways not previously associated to AS, such as cellular response to zinc, cadmium and copper ions activity (Suppl. Figure 9D, Suppl. Dataset 15).

Bibliography

1. Malone AF, et al. Rare hereditary COL4A3/COL4A4 variants may be mistaken for familial focal segmental glomerulosclerosis. *Kidney Int.* **86,** 1253-9 (2014).
2. Alsahli AA, et al. Alport's syndrome with focal segmental glomerulosclerosis lesion - Pattern to recognize. *Saudi J Kidney Dis Transpl.* **29,** 167-172 (2018).
3. Zhang P, Zhuo L, Zou Y, Li G, Peng K. *COL4A5* mutation causes Alport syndrome with focal segmental glomerulosclerosis lesion: Case report and literature review. *Clin Nephrol.* **92,** 98-102 (2019).
4. Zachara BA. Selenium and selenium-dependent antioxidants in chronic kidney disease. Adv Clin Chem. 2015;68:131-51.
5. Xu XM, Carlson BA, Mix H, Zhang Y, Saira K, et al. Biosynthesis of selenocysteine on its tRNA in eukaryotes. *PLoS Biol*. **5**, e4 (2007).
6. Sun Y, et al. Analysis of glomerular PLA2R efficacy in evaluating the prognosis of idiopathic membranous nephropathy in the background of different serum anti-PLA2R levels. *Ren Fail.* **44,** 731-740 (2022).
7. Ritchie ME, Phipson B, Wu D, Hu Y, Law CW, Shi W, Smyth GK. limma powers differential expression analyses for RNA-sequencing and microarray studies. Nucleic Acids Res. 2015 Apr 20;43(7):e47.

A

	Sample ID #	1	2	3	4	5	6	7	8	9	10	
Outlier test	ROI	AS	AS	AS	FSGS	FSGS	MN	MN	Ctrl	Ctrl	Ctrl	Total ROI
Before	Glomerular	8	9	11	3	7	6	7	12	6	6	75
	Tubular	4	3	1	2	3	3	0	12	3	0	31
After	Glomerular	8	8	11	3	7	5	7	12	6	6	73
	Tubular	3	3	1	2	3	2	0	12	3	0	29

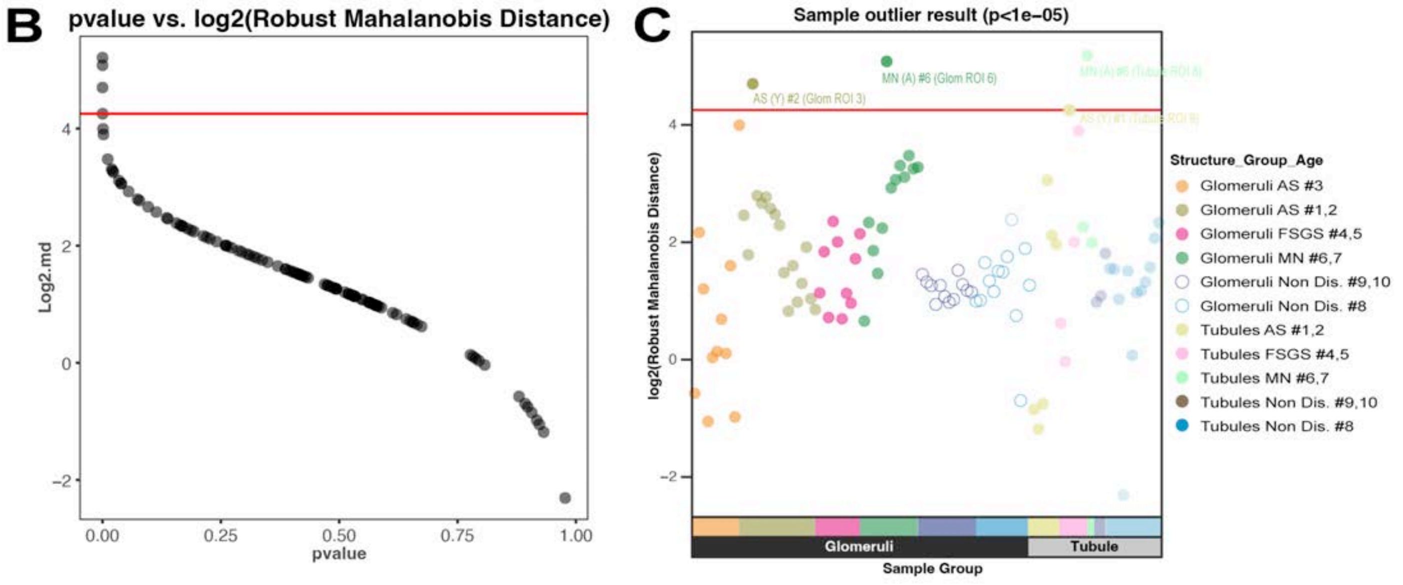

Supplementary Figure 1: Quality control assessment and elimination of glomerular and tubular ROI.

A. Summary table showing the number of glomerular and tubular ROI before and after the outlier detection for each disease and each patient based on a robust Mahalanobis distance (rMd) with a p value threshold of 0.0001 (see methods for further details). **B**. A scatter plot showing the distribution of glomerular and tubular ROI based on the log2 of the Mahalanobis distance (log2.md). The red line indicates the *P* = 0.00001 value threshold. **C**. A scatter plot showing the outlier glomerular (n=2) and tubular ROI (n=1) above the red line based on Mahalanobis distance. The red line defines the p value threshold described in (A).

Glomerular ROI from AS, FSGS, MN, and Non-Diseased biopsies

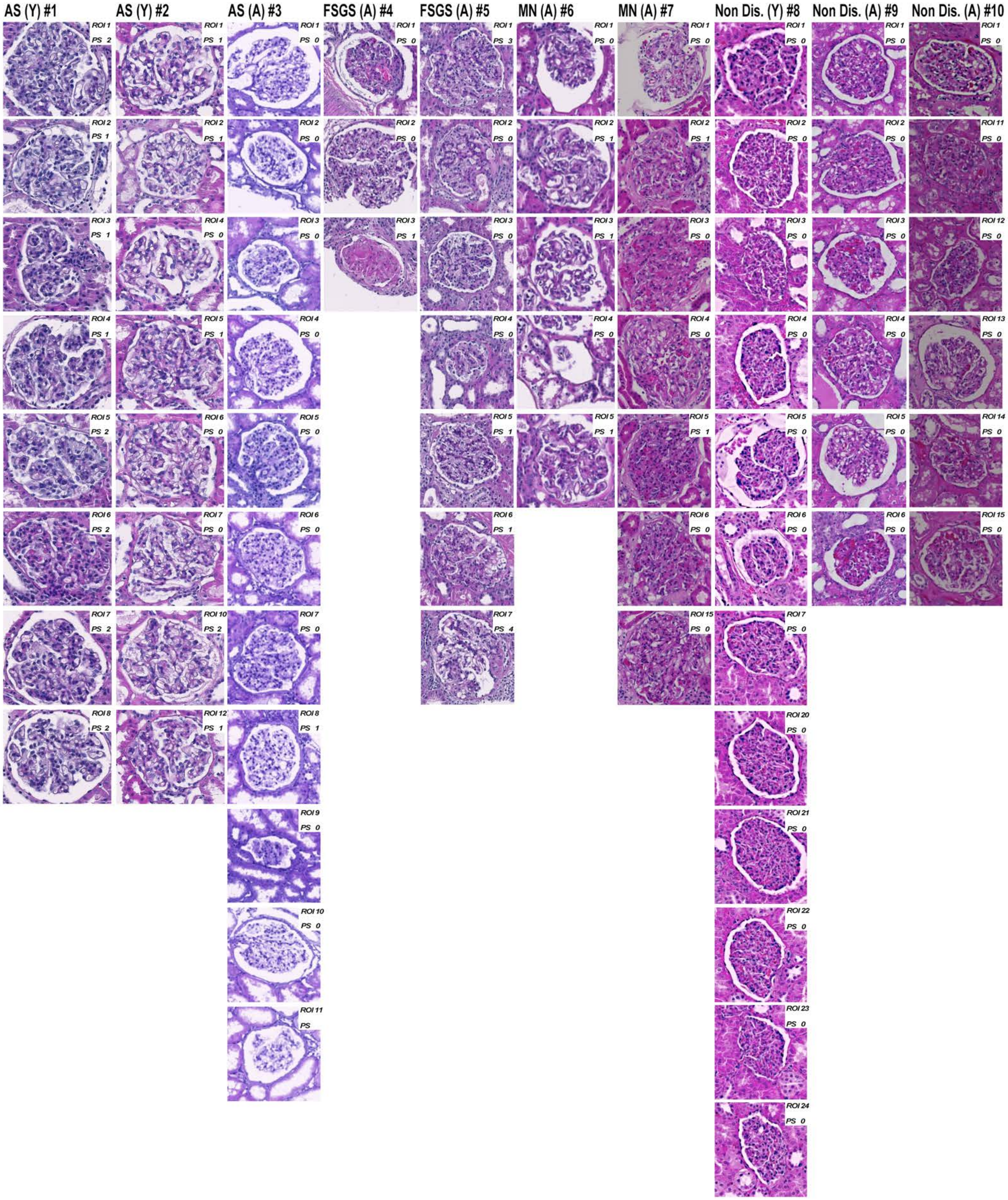

Supplementary Figure 2: Histology of all glomerular ROI.

All the kidney biopsy samples that were processed for the DSP analysis (#1-10) were stained with hematoxylin and eosin for morphological characterization and assessment blindly by a pathologist. Representative images of all the glomeruli from all the patients that passed the outlier test are shown. The number of each corresponding ROI and pathology score (PS) are shown on every glomerular image.

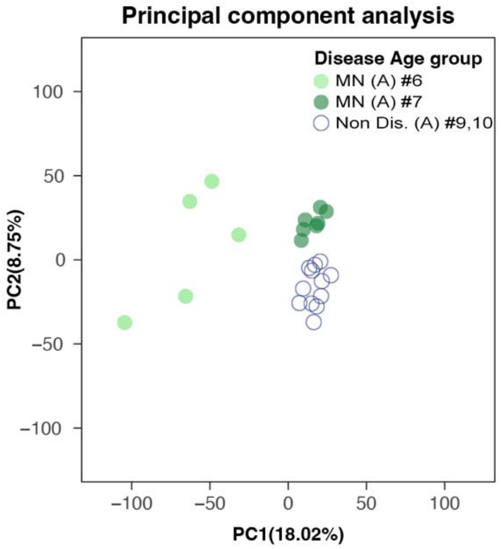

Supplementary Figure 3: Principal component analysis of MN

Unsupervised principal component analysis (PCA) based on label free quantification of the transcripts expressed in MN (#6, light green circles; #7, dark green circles), and adult non-diseased glomerular ROI (#9,10, blue circles), based on PC1 and PC2. Percentage of total variance explained by each component is indicated after each principal component.

A TOP 25 upregulated genes

GENES	AS (A) #3		FSGS (A) #4,5		MN (A) #6,7	
	log2 FC	FDR	log2 FC	FDR	log2 FC	FDR
TNS1	0.971	5.20E-05	1.318	1.42E-06	0.616	1.04E-02
CCND1	0.960	1.12E-04	0.621	5.45E-03	0.877	5.52E-03
GJA5	0.946	2.87E-03	0.867	2.74E-03	1.332	3.79E-04
UNC5B	0.937	1.17E-04	0.575	4.65E-02	0.432	4.68E-02
LAMP1	0.901	1.72E-03	0.765	4.67E-03	0.795	1.14E-02
MYO1E	0.867	4.64E-04	0.453	4.23E-02	0.718	4.39E-03
NOTCH3	0.864	1.02E-03	0.885	3.49E-05	0.653	1.19E-03
TP53INP2	0.857	2.05E-03	0.703	7.59E-05	0.442	2.42E-02
PALLD	0.811	4.60E-05	0.784	3.02E-04	0.530	3.10E-02
CLIP3	0.792	2.67E-04	0.311	2.32E-02	0.693	8.26E-03
PDGFRB	0.774	4.75E-03	0.984	3.18E-05	0.493	1.87E-02
TRIM8	0.770	1.04E-03	0.548	9.27E-03	0.663	2.68E-03
MECOM	0.736	4.60E-05	0.705	7.33E-05	0.494	3.61E-02
TM9SF2	0.731	6.67E-04	0.565	8.85E-03	0.581	8.90E-03
PRRC2B	0.727	1.81E-04	0.377	1.26E-02	0.386	8.98E-03
TBX3	0.725	3.82E-03	0.608	1.56E-02	0.768	4.39E-03
LEPROT	0.692	1.82E-03	0.536	3.23E-02	0.530	1.93E-02
DYNLRB1	0.692	9.06E-05	0.622	2.23E-04	0.904	1.55E-07
TMTC1	0.681	1.19E-02	0.914	1.43E-04	0.525	1.79E-02
RXFP1	0.655	4.03E-02	0.664	1.60E-03	0.463	2.79E-02
PLOD1	0.643	8.75E-03	0.448	3.32E-02	0.619	7.40E-03
PIM3	0.642	8.58E-03	0.515	2.22E-02	0.507	5.52E-03
PTPN12	0.633	3.05E-04	0.517	2.09E-04	0.545	7.78E-04
ZNF460	0.614	1.34E-02	0.430	9.40E-03	0.434	4.61E-02
MEIS2	0.594	2.36E-02	0.498	2.08E-02	0.705	6.04E-03

B TOP 25 downregulated genes

GENES	AS (A) #3		FSGS (A) #4,5		MN (A) #6,7	
	log2FC	FDR	log2FC	FDR	log2FC	FDR
TMEM30A	-3.605	2.48E-14	-3.805	5.13E-07	-0.907	4.50E-02
XKR6	-3.599	9.11E-15	-2.312	4.43E-11	-1.029	4.27E-05
TTC31	-3.528	1.84E-06	-3.024	1.03E-04	-1.559	3.07E-02
ADAMTS13	-3.500	1.47E-12	-4.166	3.55E-14	-1.740	1.05E-09
TMA7	-3.368	2.38E-22	-2.971	1.02E-08	-1.338	4.04E-04
HOXB8	-3.217	4.57E-10	-4.177	1.09E-18	-1.593	1.21E-08
ZNF346	-3.187	1.33E-11	-3.168	1.73E-05	-1.253	4.63E-03
TRAF3IP3	-3.175	1.04E-03	-2.384	1.96E-06	-1.818	7.28E-04
KLRD1	-3.052	3.13E-12	-3.165	1.07E-04	-1.072	2.79E-02
PLEKHN1	-2.940	1.60E-11	-3.207	2.40E-14	-0.929	2.09E-05
C1QTNF4	-2.807	1.82E-11	-2.341	2.38E-13	-0.568	1.04E-03
ZNF468	-2.780	1.21E-11	-4.780	4.98E-05	-1.535	4.12E-02
SEMA6C	-2.766	2.48E-14	-2.578	4.06E-10	-0.516	1.12E-02
INHBB	-2.661	6.73E-07	-2.644	1.31E-06	-0.883	3.41E-02
SARDH	-2.444	5.42E-12	-2.281	1.12E-02	-0.815	4.79E-02
SOX4	-2.322	4.50E-14	-2.758	1.76E-14	-0.352	3.10E-02
PSMG3	-2.274	2.64E-06	-1.985	6.39E-06	-1.002	3.06E-02
IL1RAPL2	-2.205	9.74E-13	-2.057	1.55E-09	-0.619	8.13E-04
ATP5MG	-2.161	4.25E-10	-2.179	6.56E-11	-1.057	2.19E-04
HSPB8	-2.132	1.41E-13	-4.129	1.09E-06	-0.955	5.43E-04
PRAMEF1	-2.112	1.07E-10	-2.852	8.55E-05	-0.897	3.95E-02
MAGEA4	-2.111	1.14E-01	-1.896	2.04E-03	-1.106	7.78E-04
RCHY1	-2.110	5.18E-12	-2.689	2.62E-05	-1.120	3.27E-02
CCDC13	-2.083	2.09E-04	-1.917	1.10E-04	-0.326	1.82E-02
ZNF43	-1.965	2.36E-09	-4.093	1.74E-05	-0.995	4.31E-02

Supplementary Figure 4: Top upregulated and downregulated genes common to all the adult glomeruli in AS, FSGS and MN patients.

A-B. Tables showing the 25 most highly upregulated (A) and downregulated (B) genes showing log2 fold change (log2FC), false discovery rate (FDR) for AS (#3), FSGS (#4,5) and MN (#6,7) patients compared to non-diseased controls (#9,10).

A

Neptune Glomerular Data Set	Samples
Living Donor (LD, control)	8
FSGS (focal segmental glomerulosclerosis)	93
MCD (minial change disease)	89
MN (membranous nephropathy)	62

B

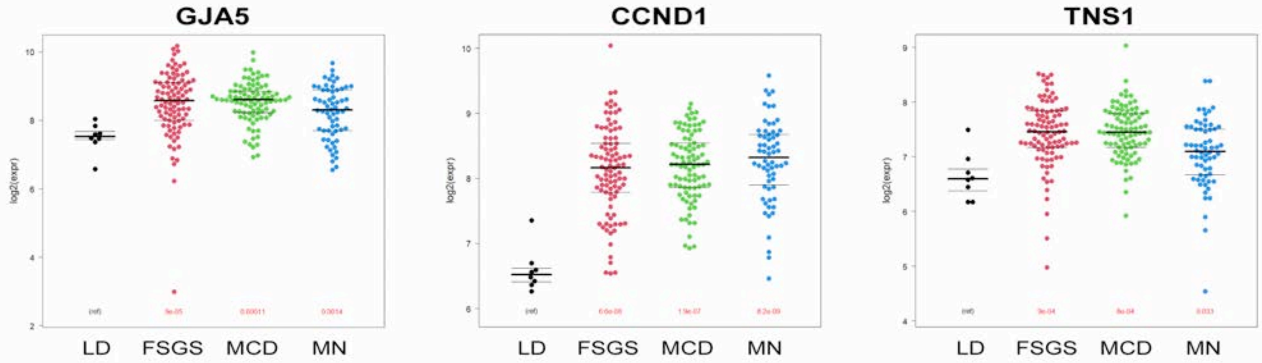

Supplementary Figure 5: Neptune glomerular data set

A. Number of samples of microdissected glomeruli from living donor (LD), FSGS, MCD (minimal change disease), and MN. Bulk RNA-seq was performed and data normalized by Limma-Voom and DE analyzed by Limma. **B**. Dot plots showing the relative expression of GJA5, CCND1, TNS1 in LD, FSGS, MCD and MN patients. P values are based on the moderated t-statistic estimated by the limma R package (7).

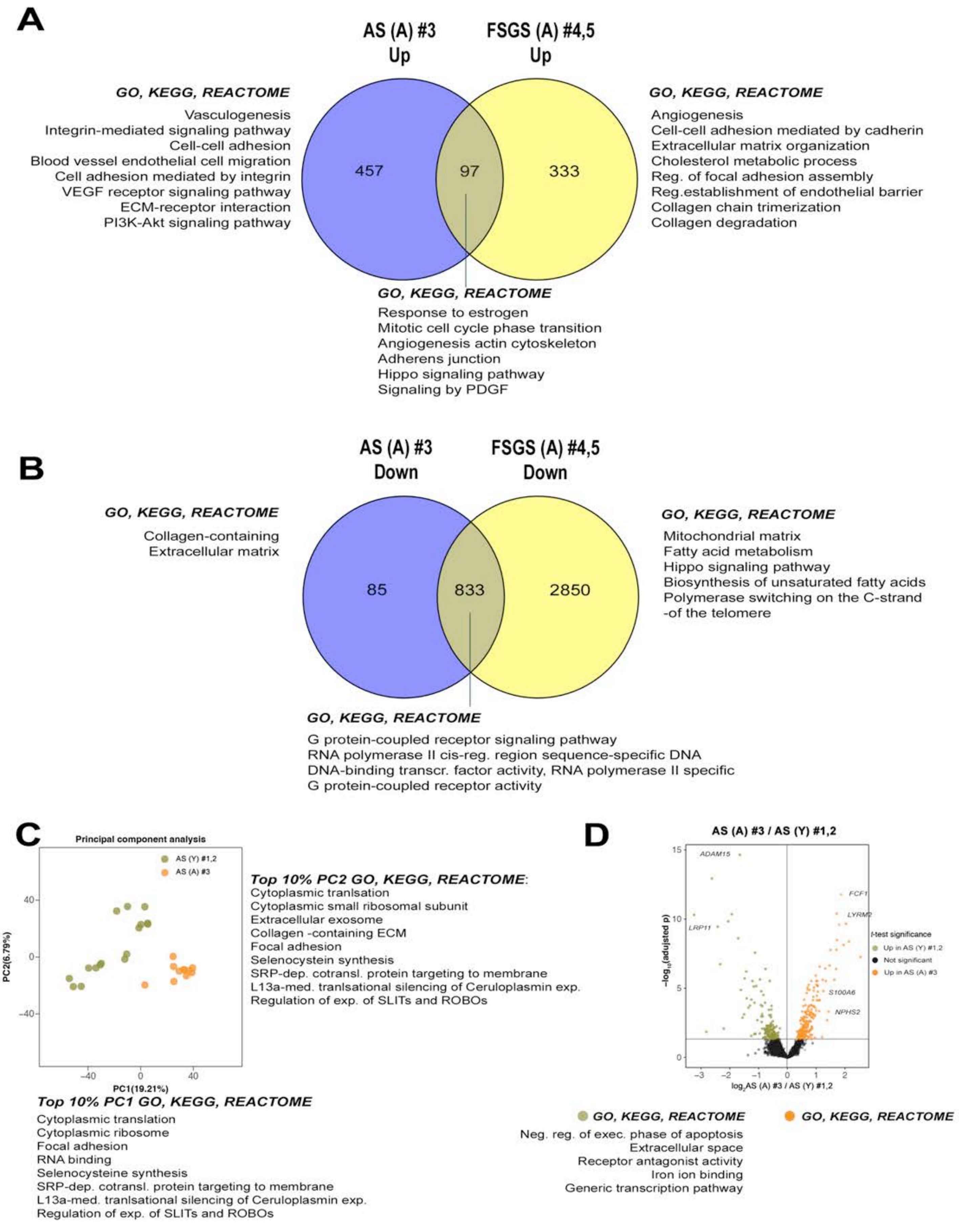
A
AS (A) #3
Up
FSGS (A) #4,5
Up
GO, KEGG, REACTOME
Vasculogenesis
Integrin-mediated signaling pathway
Cell-cell adhesion
Blood vessel endothelial cell migration
Cell adhesion mediated by integrin
VEGF receptor signaling pathway
ECM-receptor interaction
PI3K-Akt signaling pathway
457
97
333
GO, KEGG, REACTOME
Angiogenesis
Cell-cell adhesion mediated by cadherin
Extracellular matrix organization
Cholesterol metabolic process
Reg. of focal adhesion assembly
Reg.establishment of endothelial barrier
Collagen chain trimerization
Collagen degradation
GO, KEGG, REACTOME
Response to estrogen
Mitotic cell cycle phase transition
Angiogenesis actin cytoskeleton
Adherens junction
Hippo signaling pathway
Signaling by PDGF
B
AS (A) #3
Down
FSGS (A) #4,5
Down
GO, KEGG, REACTOME
Collagen-containing
Extracellular matrix
85
833
2850
GO, KEGG, REACTOME
Mitochondrial matrix
Fatty acid metabolism
Hippo signaling pathway
Biosynthesis of unsaturated fatty acids
Polymerase switching on the C-strand
-of the telomere
GO, KEGG, REACTOME
G protein-coupled receptor signaling pathway
RNA polymerase II cis-reg. region sequence-specific DNA
DNA-binding transcr. factor activity, RNA polymerase II specific
G protein-coupled receptor activity
C
Principal component analysis
AS (Y) #1,2
AS (A) #3
PC2(6.79%)
PC1(19.21%)
Top 10% PC2 GO, KEGG, REACTOME:
Cytoplasmic tranlsation
Cytoplasmic small ribosomal subunit
Extracellular exosome
Collagen -containing ECM
Focal adhesion
Selenocystein synthesis
SRP-dep. cotransl. protein targeting to membrane
L13a-med. tranlsational silencing of Ceruloplasmin exp.
Regulation of exp. of SLITs and ROBOs
Top 10% PC1 GO, KEGG, REACTOME
Cytoplasmic translation
Cytoplasmic ribosome
Focal adhesion
RNA binding
Selenocysteine synthesis
SRP-dep. cotransl. protein targeting to membrane
L13a-med. tranlsational silencing of Ceruloplasmin exp.
Regulation of exp. of SLITs and ROBOs
D
AS (A) #3 / AS (Y) #1,2
ADAM15
LRP11
FCF1
LYRM2
S100A6
NPHS2
t-test significance
Up in AS (Y) #1,2
Not significant
Up in AS (A) #3
GO, KEGG, REACTOME
GO, KEGG, REACTOME
Neg. reg. of exec. phase of apoptosis
Extracellular space
Receptor antagonist activity
Iron ion binding
Generic transcription pathway

Supplementary Figure 6: Transcriptional program of glomeruli in adult AS compared with adult FSGS and young AS.

A. Venn diagram showing the total number of differentially upregulated genes (Student t-test, and binomial test BH adjusted-*P* < 0.05) in AS (#3) and FSGS (#4-5). A list of significantly enriched GO terms, KEGG and REACTOME pathways (EASE modified Fisher's exact p<0.05) for the genes commonly upregulated (n=97 genes), upregulated only in AS (#3, n=457) and only in FSGS (#4,5, n=333) are depicted next to the Venn diagram. **B**. Venn diagram showing the total number of differentially downregulated genes (Student t-test, and binomial test BH adjusted-*P* < 0.05) in AS (#3) and FSGS (#4-5). A list of significantly enriched GO terms, KEGG and REACTOME pathways (EASE modified Fisher's exact p<0.05) for the genes commonly downregulated (n=833 genes), downregulated only in AS (#3, n=85) and only in FSGS (#4,5, n=2,850) are depicted next to the Venn diagram. **C.** Unsupervised principal component analysis based on label free quantification of the transcripts expressed in glomeruli of AS (#3) versus AS (#1,2) patients analyzed based on principal components PC1 and PC2. Percentage of total variance is indicated on each PC axis. A list of significantly enriched GO terms, KEGG and REACTOME pathways (EASE modified Fisher's exact p<0.05) for the list of 10% transcripts significantly higher or lower in abundance in each PC are shown next to the plot. **D.** Volcano plot representing the result of the Student's *t*-test (BH adjusted-*P* < 0.05) comparison of differential gene expression between glomeruli of AS (#3) versus AS (#1,2) patients. A select list of GO terms, and KEGG pathways significantly enriched (EASE modified Fisher exact, *P* < 0.05) for genes expressed higher in each group (Student's t-test and binomial test) is depicted on each side of the volcano plot.

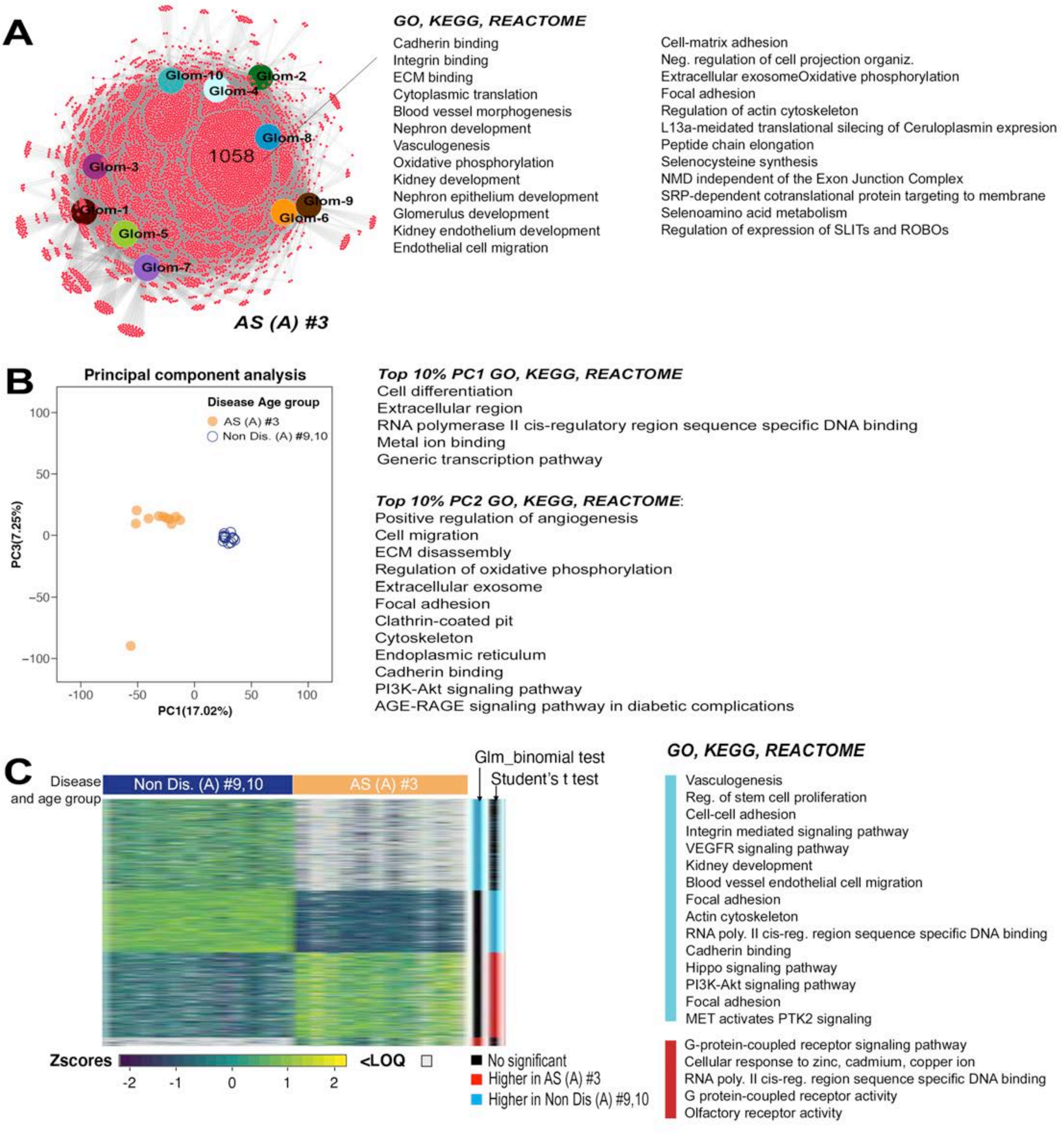
A
Glom-10
Glom-2
Glom-4
Glom-8
1058
Glom-3
Glom-1
Glom-9
Glom-6
Glom-5
Glom-7
AS (A) #3
GO, KEGG, REACTOME
Cadherin binding
Integrin binding
ECM binding
Cytoplasmic translation
Blood vessel morphogenesis
Nephron development
Vasculogenesis
Oxidative phosphorylation
Kidney development
Nephron epithelium development
Glomerulus development
Kidney endothelium development
Endothelial cell migration
Cell-matrix adhesion
Neg. regulation of cell projection organiz.
Extracellular exosomeOxidative phosphorylation
Focal adhesion
Regulation of actin cytoskeleton
L13a-meidated translational silecing of Ceruloplasmin expresion
Peptide chain elongation
Selenocysteine synthesis
NMD independent of the Exon Junction Complex
SRP-dependent cotranslational protein targeting to membrane
Selenoamino acid metabolism
Regulation of expression of SLITs and ROBOs
B
Principal component analysis
Disease Age group
AS (A) #3
Non Dis. (A) #9,10
100
50
0
−50
−100
PC3(7.25%)
-100
-50
0
50
100
PC1(17.02%)
Top 10% PC1 GO, KEGG, REACTOME
Cell differentiation
Extracellular region
RNA polymerase II cis-regulatory region sequence specific DNA binding
Metal ion binding
Generic transcription pathway
Top 10% PC2 GO, KEGG, REACTOME:
Positive regulation of angiogenesis
Cell migration
ECM disassembly
Regulation of oxidative phosphorylation
Extracellular exosome
Focal adhesion
Clathrin-coated pit
Cytoskeleton
Endoplasmic reticulum
Cadherin binding
PI3K-Akt signaling pathway
AGE-RAGE signaling pathway in diabetic complications
C
Disease
and age group
Non Dis. (A) #9,10
AS (A) #3
Glm_binomial test
Student's t test
Zscores
-2
-1
0
1
2
<LOQ
No significant
Higher in AS (A) #3
Higher in Non Dis (A) #9,10
GO, KEGG, REACTOME
Vasculogenesis
Reg. of stem cell proliferation
Cell-cell adhesion
Integrin mediated signaling pathway
VEGFR signaling pathway
Kidney development
Blood vessel endothelial cell migration
Focal adhesion
Actin cytoskeleton
RNA poly. II cis-reg. region sequence specific DNA binding
Cadherin binding
Hippo signaling pathway
PI3K-Akt signaling pathway
Focal adhesion
MET activates PTK2 signaling
G-protein-coupled receptor signaling pathway
Cellular response to zinc, cadmium, copper ion
RNA poly. II cis-reg. region sequence specific DNA binding
G protein-coupled receptor activity
Olfactory receptor activity

Supplementary Figure 7: Gene expression signature in adult AS glomeruli.

A. Venn diagram of TMM normalized expression of transcripts detected above LOQ in the glomeruli (n=11) of adult AS patient (#3; note the Venn diagram is showing the maximum of 10 gloms), showing the distribution of the number of genes commonly expressed in all AS #3 glomeruli (n= 1,058 genes). The black line points to the enriched pathways (GO, KEGG, REACTOME) for the commonly expressed genes between all glomeruli. **B**. Unsupervised principal component analysis (PCA) based on label free quantification of the transcripts expressed in young AS (#3, orange circles), and adult non-diseased glomerular ROI (#9,10, blue circles), based on PC1 and PC2. Percentage of variance explained by each principal component is indicated after the principal components. A select list of significantly enriched GO terms, KEGG and REACTOME pathways (EASE modified Fisher's exact $p<0.05$) for the list of 10% transcripts significantly higher or lower in abundance in PC1 and PC2 are shown next to the plot. **C**. Heatmap depicting the transcripts significantly modulated between AS (#3), and non-diseased (#9,10); analysis of variance Student's t-test and binomial test, BH adjusted-$P < 0.05$. Transcripts less than LOQ in value are shown in white. A select list of GO terms, KEGG and REACTOME pathways significantly enriched (EASE modified Fisher exact, $P < 0.05$) for the upregulated and downregulated genes in AS (#3) versus non-diseased (#9,10) are shown to the right of the heatmap.

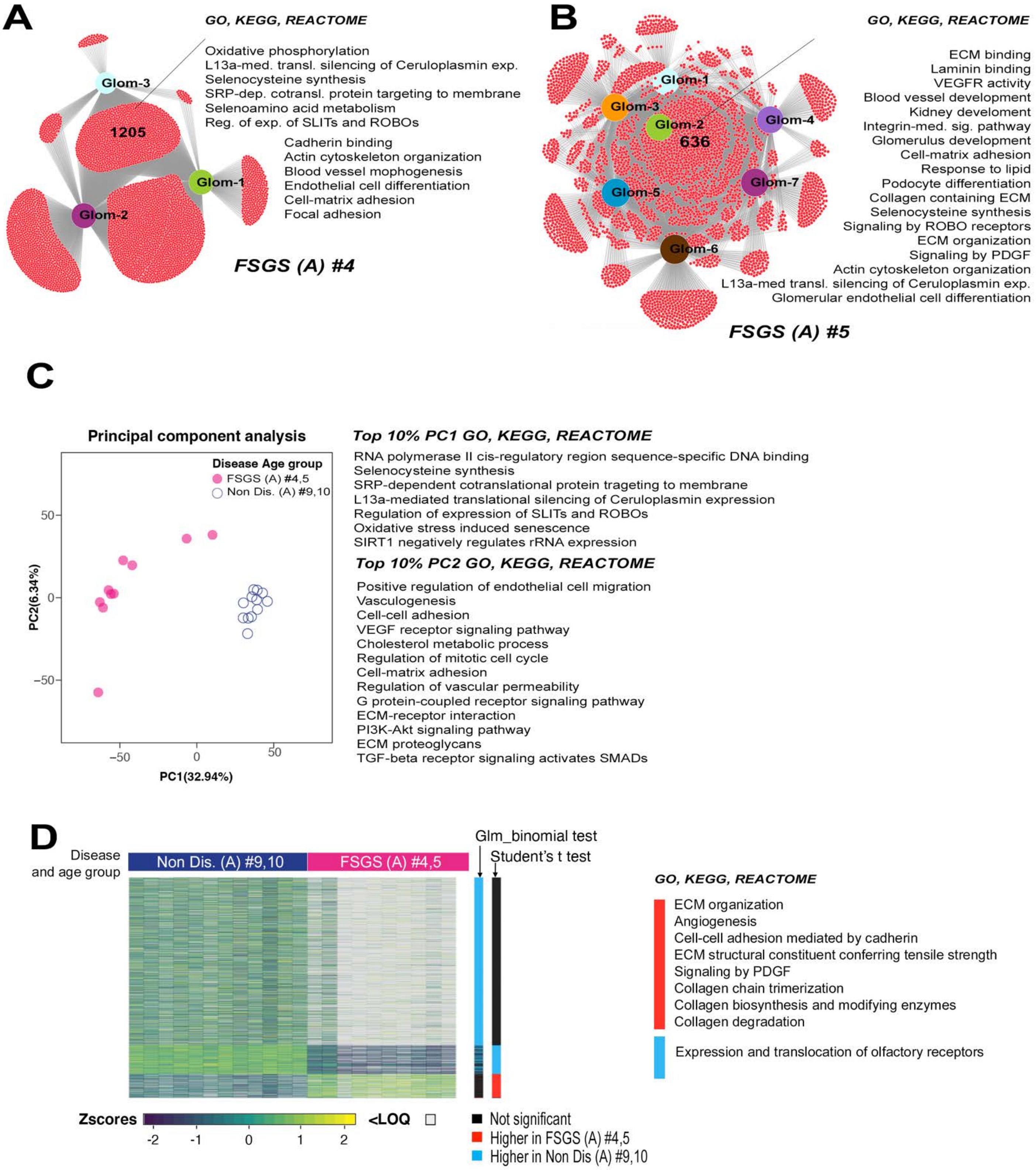

A
GO, KEGG, REACTOME
Oxidative phosphorylation
L13a-med. transl. silencing of Ceruloplasmin exp.
Selenocysteine synthesis
SRP-dep. cotransl. protein targeting to membrane
Selenoamino acid metabolism
Reg. of exp. of SLITs and ROBOs
Cadherin binding
Actin cytoskeleton organization
Blood vessel mophogenesis
Endothelial cell differentiation
Cell-matrix adhesion
Focal adhesion
Glom-3
1205
Glom-1
Glom-2
FSGS (A) #4
B
GO, KEGG, REACTOME
ECM binding
Laminin binding
VEGFR activity
Blood vessel development
Kidney develoment
Integrin-med. sig. pathway
Glomerulus development
Cell-matrix adhesion
Response to lipid
Podocyte differentiation
Collagen containing ECM
Selenocysteine synthesis
Signaling by ROBO receptors
ECM organization
Signaling by PDGF
Actin cytoskeleton organization
L13a-med transl. silencing of Ceruloplasmin exp.
Glomerular endothelial cell differentiation
Glom-1
Glom-3
Glom-2
636
Glom-4
Glom-7
Glom-5
Glom-6
FSGS (A) #5
C
Principal component analysis
Disease Age group
FSGS (A) #4,5
Non Dis. (A) #9,10
50
0
−50
PC2(6.34%)
−50
0
50
PC1(32.94%)
Top 10% PC1 GO, KEGG, REACTOME
RNA polymerase II cis-regulatory region sequence-specific DNA binding
Selenocysteine synthesis
SRP-dependent cotranslational protein trageting to membrane
L13a-mediated translational silencing of Ceruloplasmin expression
Regulation of expression of SLITs and ROBOs
Oxidative stress induced senescence
SIRT1 negatively regulates rRNA expression
Top 10% PC2 GO, KEGG, REACTOME
Positive regulation of endothelial cell migration
Vasculogenesis
Cell-cell adhesion
VEGF receptor signaling pathway
Cholesterol metabolic process
Regulation of mitotic cell cycle
Cell-matrix adhesion
Regulation of vascular permeability
G protein-coupled receptor signaling pathway
ECM-receptor interaction
PI3K-Akt signaling pathway
ECM proteoglycans
TGF-beta receptor signaling activates SMADs
D
Disease
and age group
Non Dis. (A) #9,10
FSGS (A) #4,5
Glm_binomial test
Student's t test
GO, KEGG, REACTOME
ECM organization
Angiogenesis
Cell-cell adhesion mediated by cadherin
ECM structural constituent conferring tensile strength
Signaling by PDGF
Collagen chain trimerization
Collagen biosynthesis and modifying enzymes
Collagen degradation
Expression and translocation of olfactory receptors
Zscores
-2
-1
0
1
2
<LOQ
Not significant
Higher in FSGS (A) #4,5
Higher in Non Dis (A) #9,10

Supplementary Figure 8: Gene expression signature in adult FSGS glomeruli.

A-B. Venn diagrams of TMM normalized expression of genes detected above LOQ in the adult FSGS biopsies (sample #4,5), showing the distribution of the number of genes commonly expressed in FSGS #4 glomeruli (n=1,205 genes; A), and FSGS #5 (n=656 genes; B). The black lines point to the enriched pathways (GO, KEGG, REACTOME) for the commonly expressed genes in FSGS #4 and #5. **C**. Unsupervised principal component analysis (PCA) based on label free quantification of the transcripts expressed in adult FSGS (#4,5, pink circles), and adult non-diseased glomerular ROI (#9,10, blue circles), based on PC1 and PC2. Percentage of variance explained by each principal component is indicated after the principal components. A select list of significantly enriched GO terms, KEGG and REACTOME pathways (EASE modified Fisher's exact $p<0.05$) for the list of 10% transcripts significantly higher or lower in abundance in PC1 and PC2 are shown next to the plot. **D**. Heatmap depicting the transcripts significantly modulated between FSGS (#4,5), and non-diseased (#9,10); analysis of variance Student's t-test and binomial test, BH-adjusted $P < 0.05$. Transcripts less than LOQ in value are shown in white. A selection of GO terms, KEGG and REACTOME pathways significantly enriched (EASE modified Fisher exact, $P < 0.05$) for the upregulated and downregulated genes in FSGS (#4,5) versus non-diseased (#9,10) are shown to the right of the heatmap.

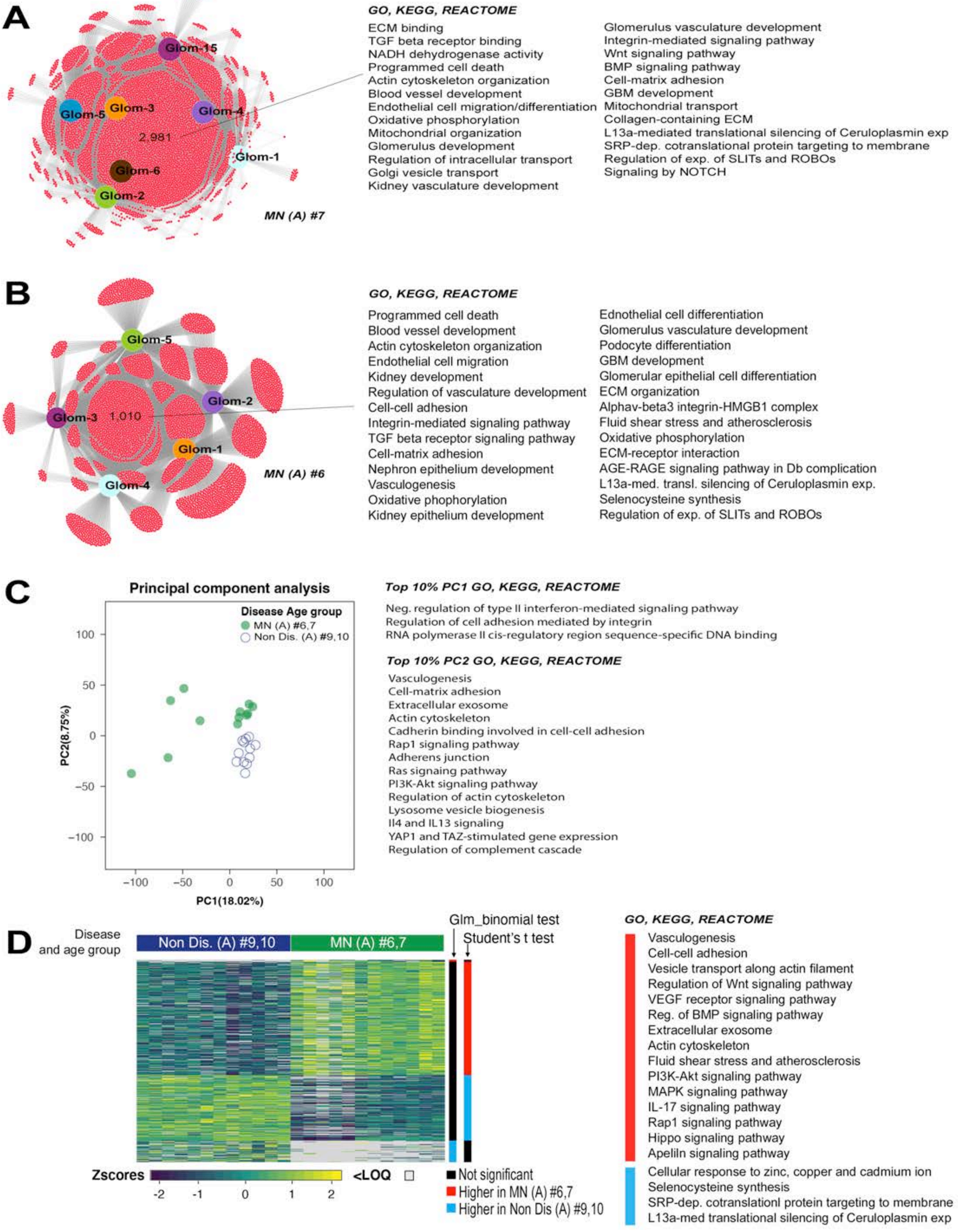
A
GO, KEGG, REACTOME
ECM binding
TGF beta receptor binding
NADH dehydrogenase activity
Programmed cell death
Actin cytoskeleton organization
Blood vessel development
Endothelial cell migration/differentiation
Oxidative phosphorylation
Mitochondrial organization
Glomerulus development
Regulation of intracellular transport
Golgi vesicle transport
Kidney vasculature development
Glomerulus vasculature development
Integrin-mediated signaling pathway
Wnt signaling pathway
BMP signaling pathway
Cell-matrix adhesion
GBM development
Mitochondrial transport
Collagen-containing ECM
L13a-mediated translational silencing of Ceruloplasmin exp
SRP-dep. cotranslational protein targeting to membrane
Regulation of exp. of SLITs and ROBOs
Signaling by NOTCH
Glom-15
Glom-5
Glom-3
Glom-4
2,981
Glom-1
Glom-6
Glom-2
MN (A) #7
B
GO, KEGG, REACTOME
Programmed cell death
Blood vessel development
Actin cytoskeleton organization
Endothelial cell migration
Kidney development
Regulation of vasculature development
Cell-cell adhesion
Integrin-mediated signaling pathway
TGF beta receptor signaling pathway
Cell-matrix adhesion
Nephron epithelium development
Vasculogenesis
Oxidative phophorylation
Kidney epithelium development
Ednothelial cell differentiation
Glomerulus vasculature development
Podocyte differentiation
GBM development
Glomerular epithelial cell differentiation
ECM organization
Alphav-beta3 integrin-HMGB1 complex
Fluid shear stress and atherosclerosis
Oxidative phosphorylation
ECM-receptor interaction
AGE-RAGE signaling pathway in Db complication
L13a-med. transl. silencing of Ceruloplasmin exp.
Selenocysteine synthesis
Regulation of exp. of SLITs and ROBOs
Glom-5
Glom-2
Glom-3
1,010
Glom-1
Glom-4
MN (A) #6
C
Principal component analysis
Disease Age group
MN (A) #6,7
Non Dis. (A) #9,10
PC2(8.75%)
PC1(18.02%)
100
50
0
−50
−100
−100
−50
0
50
100
Top 10% PC1 GO, KEGG, REACTOME
Neg. regulation of type II interferon-mediated signaling pathway
Regulation of cell adhesion mediated by integrin
RNA polymerase II cis-regulatory region sequence-specific DNA binding
Top 10% PC2 GO, KEGG, REACTOME
Vasculogenesis
Cell-matrix adhesion
Extracellular exosome
Actin cytoskeleton
Cadherin binding involved in cell-cell adhesion
Rap1 signaling pathway
Adherens junction
Ras signaing pathway
PI3K-Akt signaling pathway
Regulation of actin cytoskeleton
Lysosome vesicle biogenesis
Il4 and IL13 signaling
YAP1 and TAZ-stimulated gene expression
Regulation of complement cascade
D
Disease
and age group
Non Dis. (A) #9,10
MN (A) #6,7
Glm_binomial test
Student's t test
Zscores
-2
-1
0
1
2
<LOQ
Not significant
Higher in MN (A) #6,7
Higher in Non Dis (A) #9,10
GO, KEGG, REACTOME
Vasculogenesis
Cell-cell adhesion
Vesicle transport along actin filament
Regulation of Wnt signaling pathway
VEGF receptor signaling pathway
Reg. of BMP signaling pathway
Extracellular exosome
Actin cytoskeleton
Fluid shear stress and atherosclerosis
PI3K-Akt signaling pathway
MAPK signaling pathway
IL-17 signaling pathway
Rap1 signaling pathway
Hippo signaling pathway
Apeliln signaling pathway
Cellular response to zinc, copper and cadmium ion
Selenocysteine synthesis
SRP-dep. cotranslationl protein targeting to membrane
L13a-med translational silencing of Ceruloplasmin exp

Supplementary Figure 9: Gene expression signature in adult MN glomeruli.

A-B. Venn diagrams of TMM normalized expression of genes detected above LOQ in the adult MN biopsies (sample #6,7), showing the distribution of the number of genes commonly expressed in MN #7 glomeruli (n=2,981 genes; A), and MN #6 (n=1,010 genes; B). The black lines point to the enriched pathways (GO, KEGG, REACTOME) for the commonly expressed genes in MN #7 (A) and #6 (B). **C**. Unsupervised principal component analysis (PCA) based on label free quantification of the transcripts expressed in adult MN (#6,7 green), and adult non-diseased glomerular ROI (#9,10, blue circles), based on PC1 and PC2. Percentage of explained variance is indicated after each principal component. A select list of significantly enriched GO terms, KEGG and REACTOME pathways (EASE modified Fisher's exact $p<0.05$) for the list of the top 10% transcripts contributing to PC1 and PC2 are shown next to the plot. **D**. Heatmap depicting the transcripts significantly modulated between MN (#6,7), and non-diseased (#9,10); analysis of variance Student's t-test and binomial test, BH adjusted-$P < 0.05$. Transcripts less than LOQ in value are shown in white. A select list of GO terms, KEGG and REACTOME pathways significantly enriched (EASE modified Fisher exact, $P < 0.05$) for the upregulated and downregulated genes in MN (#6,7) versus non-diseased (#9,10) are shown to the right of the heatmap.

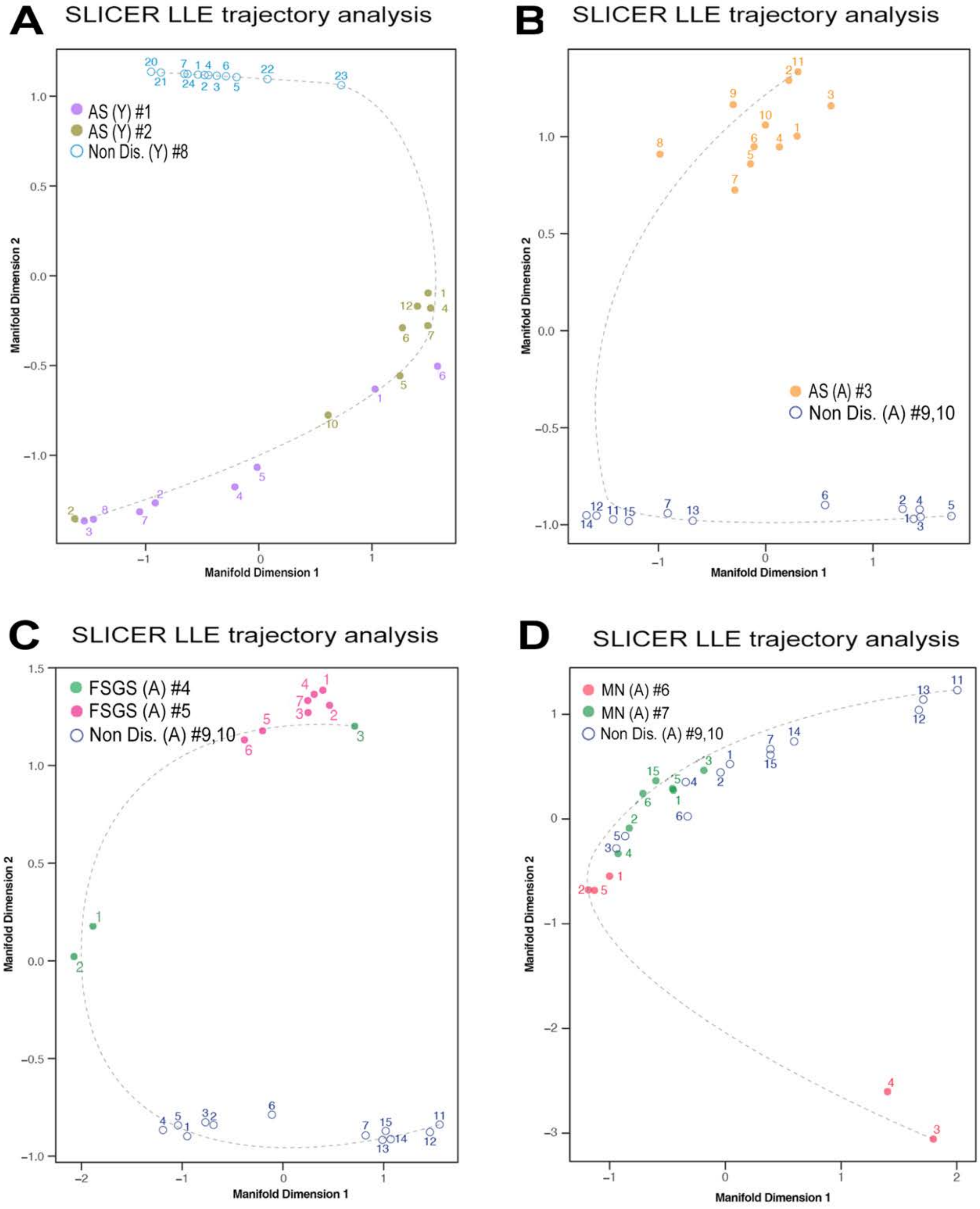

Supplementary Figure 10: Trajectory analysis.

A-F. Mapping of gene expression perturbation data to the inferred trajectories by SLICER from non-diseased control (#8, blue) and young AS (#1 purple, #2 grey-green; A), non-diseased control (#9-10) and adult AS (#3, orange; B), adult FSGS (#4 green, #5 pink; C), adult MN (#6 red, #7, green; D). The number of the glomerular ROI are depicted next to each data point. The dotted lines represent fitting curves that indicate the relationship between different glomeruli on the trajectory path.

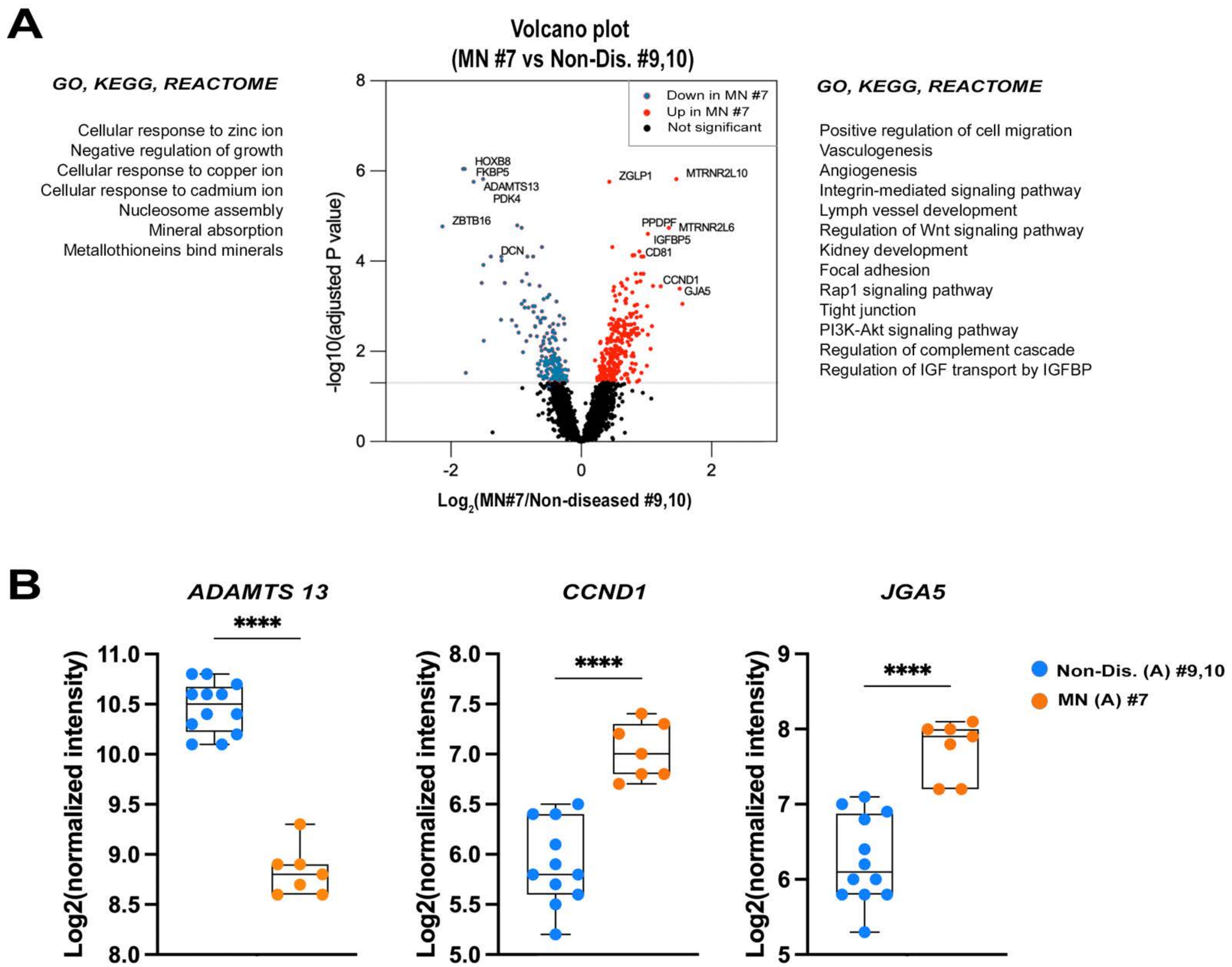

Supplementary Figure 11: Glomerular expression of *ADAMTS13*, *CCND1* and *GJA5* in PLA2R positive MN patient. A. Volcano plot showing the transcript modulated in MN versus non-diseased controls, Student's *t*-test BH adjusted-*P* were used to generate this plot. A select list of GO terms, and KEGG pathways significantly enriched (EASE modified Fisher exact, $P < 0.05$) for genes expressed higher in each group (Student's t-test and binomial test) is depicted on each side of the volcano plot. Boxplots of normalized intensities for *ADAMTS13, CCND1, GJA5* in glomeruli of PLA2R positive MN patient #7 versus non-diseased control (#9,10). Each dot represents a glomerular ROI. Student's *t*-test ****P < 0.0001.
